# A sulfotyrosine motif in the human TrkB extracellular domain is required for activation by the agonist antibody ZEB85

**DOI:** 10.1073/pnas.2618780123

**Published:** 2026-10-29

**Authors:** David C. Briggs, Ryan T. Duffy, Sarah Ateaque, Sarah Maslen, Hema Nagaraj, Yves-Alain Barde, Peter S. DiStefano, Ronald M. Lindsay, Paul C. Armstrong, Chloe J. Peach, Neil Q. McDonald

**Affiliations:** ^a^ Signalling and Structural Biology Lab, The Francis Crick Institute, London NW1 1AT, United Kingdom; ^b^ Centre of Membrane Proteins and Receptors, School of Life Sciences, University of Nottingham, Nottingham NG7 2UH, United Kingdom; ^c^ School of Biosciences, Cardiff University, Cardiff CF10 3AX, Wales, United Kingdom; ^d^ Proteomics Science Technology Platform, The Francis Crick Institute, London NW1 1AT, United Kingdom; ^e^ Chemical Biology Science Technology Platform, The Francis Crick Institute, London NW1 1AT, United Kingdom; ^f^Zebra Biologics Inc, Concord, MA 01742; ^g^ Blizard Institute, The Faculty of Medicine and Dentistry, Queen Mary University of London, London E1 2AT, United Kingdom; ^h^ Institute of Structural and Molecular Biology, School of Natural Sciences, Birkbeck College, London WC1E 7HX, United Kingdom

**Keywords:** neurotrophins, BDNF, TrkB, agonist, sulfotyrosine

## Abstract

The brain-derived neurotrophic factor (BDNF)-tropomyosin receptor kinase B (TrkB) signaling axis is a key effector of synaptic plasticity and neuroprotection. While TrkB activation is a major target to prevent neural dysfunction, stimulation with exogenous BDNF administration has not proven tractable given the unfavorable physiochemical properties of BDNF. In addition, BDNF also activates a cell death pathway by binding to the neurotrophin receptor p75. The TrkB agonist antibody ZEB85 provides an alternative route to the selective activation of TrkB. To understand the ZEB85 mechanism of action, we mapped the binding site for ZEB85 to the TrkB extracellular juxtamembrane domain and found a sulfotyrosine modification at residue 400 crucial for ZEB85 interaction and activation of TrkB signaling. We determined the structures of ZEB85 and BDNF bound to their respective binding sites on human TrkB suggesting independent sites for each ligand, despite prior evidence of direct competition. Using structure-guided BDNF- and ZEB85-binding deficient TrkB mutants, we assessed their ability to sequester ligands from full-length TrkB in cultured human neurons. We found that the BDNF binding site extends into the juxtamembrane domain of TrkB but does not require the sulfotyrosine 400 to activate TrkB. Together with biophysical analysis and AlphaFold modeling, these results explain how BDNF can displace ZEB85 from TrkB through an overlapping epitope. Our findings reveal unique features of TrkB, not present in the related neurotrophin receptors TrkA and TrkC and suggest future directions to explore the role of sulfotyrosine in TrkB signaling.

## Significance

Binding of brain-derived neurotrophic factor (BDNF) to tropomyosin receptor kinase B (TrkB) promotes signaling cascades critical for neuronal survival and synaptic plasticity. Potent TrkB agonist antibodies have been identified with superior handling properties to BDNF, but how they mimic BDNF so closely is not known. This study uncovers an unexpected sulfotyrosine modification in the extracellular juxtamembrane domain of human TrkB crucial for activation by the agonist antibody, ZEB85. The sulfotyrosine motif is unique to TrkB and not TrkA or TrkC receptors. The study shows that BDNF also binds to this domain but does so independently of the sulfotyrosine motif, accounting for how BDNF and ZEB85 can compete for TrkB. Our findings enable the design of next-generation modulators of TrkB activation.

The tropomyosin-related kinase B (TrkB) is a key signaling receptor tyrosine kinase for the brain-derived neurotrophic factor (BDNF) ligand (1). TrkB is expressed widely in the CNS and is a key target for neuroprotection in neurodegenerative disease. BDNF signaling through TrkB plays a crucial role in the synaptic regulation of brain physiology and pathology including neuronal differentiation (2), synaptogenesis (3), neuronal architecture (4), synaptic plasticity (5), depression (6), and neurodegeneration (1). Enthusiasm for BDNF therapy, despite showing promise in cellular and animal-models of neurodegeneration (7), has been tempered by inconsistent findings and complex underlying mechanisms of its action, and the poor bioavailability of BDNF. In addition to this, proBDNF and mature BDNF show affinity toward the p75NTR receptor which has a quite distinct signaling pathway driving neuronal apoptosis (8). In contrast, recent attention has focused on agonists of TrkB in different formats such as small molecules (9, 10) peptides (11), and antibodies (12–15). Among several reported TrkB antibody-based agonists is the full TrkB agonist ZEB85 (a fully human sc-Fv-Fc fusion protein) identified by coupling a function-based cellular assay to screening a combinatorial scFv library (15). Characterization of ZEB85 using a human TrkB reporter cell line and BDNF-responsive GABAergic neurons, showed that ZEB85 is a high-fidelity agonist of TrkB with comparable autophosphorylation, signaling, and transcriptional responses to BDNF (15). Furthermore, ZEB85 binding to TrkB was shown to be displaced by BDNF in a flow-cytometry based competition assay, suggesting overlap in their respective TrkB binding epitopes (15).

The TrkB extracellular module shares the same domain composition as TrkA and TrkC, comprising a leucine-rich repeat domain (LRR), two consecutive Ig domains (IG1-IG2), and a more divergent extracellular juxtamembrane region (eJM) prior to the transmembrane helix. Insights from structures of the TrkA–NGF complex (16) and TrkB-NT4/5 heterodimer (17) have demonstrated the 2nd IG domain of Trk receptors contains the key neurotrophin-binding determinants, with recent studies also suggesting the extracellular module is coupled to kinase activation through less well-defined regions such as the eJM (18).

The TrkB receptor extracellular module is known to be extensively glycosylated (19), but the heterogeneous nature of glycosylation can make other posttranslational modifications hard to detect. Sulfotyrosine (sY or sTyr) is a relatively uncommon modification of extracellular proteins, first isolated in fibrinogen (20). Detection of sulfotyrosine residues has historically been quite difficult, as the sulfo group is not only smaller than the degree of heterogeneity typically observed in glycan modifications, but is also labile in strong acids that are used in Edman degradation and routine mass spectrometry protocols (21). Recent advances have led to protocols being developed to overcome this issue (22). Since no “eraser” activities have been found in mammals, sulfotyrosine is stable in most cellular environments (except for strong acids) and is considered an irreversible modification (23). In mammals, this tyrosine modification is “written” by one of two tyrosyl protein sulfotransferases (TPST1/2). TPST enzymes are single-pass type I transmembrane proteins with their catalytic domain located in the lumen of the trans-Golgi, able to transfer a sulfate group from a PAPS (3′-phosphoadenosine 5′-phosphosulfate) cofactor to the hydroxyl group of a tyrosine residue (24). Structure–function analysis of human TSPT1 and 2, and analysis of known sulfotyrosine-containing proteins shows a preference for acidic amino acids in close proximity to the target tyrosine residue(s) (25, 26). Extracellular proteins within the complement and coagulation pathways are frequently modified by sulfotyrosine and modifications are frequently found in or adjacent to protein–protein interaction sites where they contribute to the interaction surface and enhance binding affinity (27).

To understand the mechanism of action for ZEB85 (and other TrkB agonists) and its close mimicry of BDNF in terms of signaling and transcriptional responses, we mapped the ZEB85 epitope on TrkB. We uncovered an unexpected sulfotyrosine modification in human TrkB that is required for ZEB85 binding and full agonist activity as compared to BDNF. This epitope lies within an extracellular juxtamembrane region that is highly divergent between TrkA and TrkC receptor isoforms. We determined ZEB85 and BDNF structures bound to their respective TrkB binding epitopes including the first report of the structure of a TrkB extracellular domain-BDNF complex. We then used complementary biophysical and competition assays to reveal that both ZEB85 and BDNF interact with the TrkB extracellular juxtamembrane region.

## Results

### Mapping the ZEB85 Epitope and Identification of a Sulfotyrosine Modification of TrkB eJM.

**Fig. 1.**
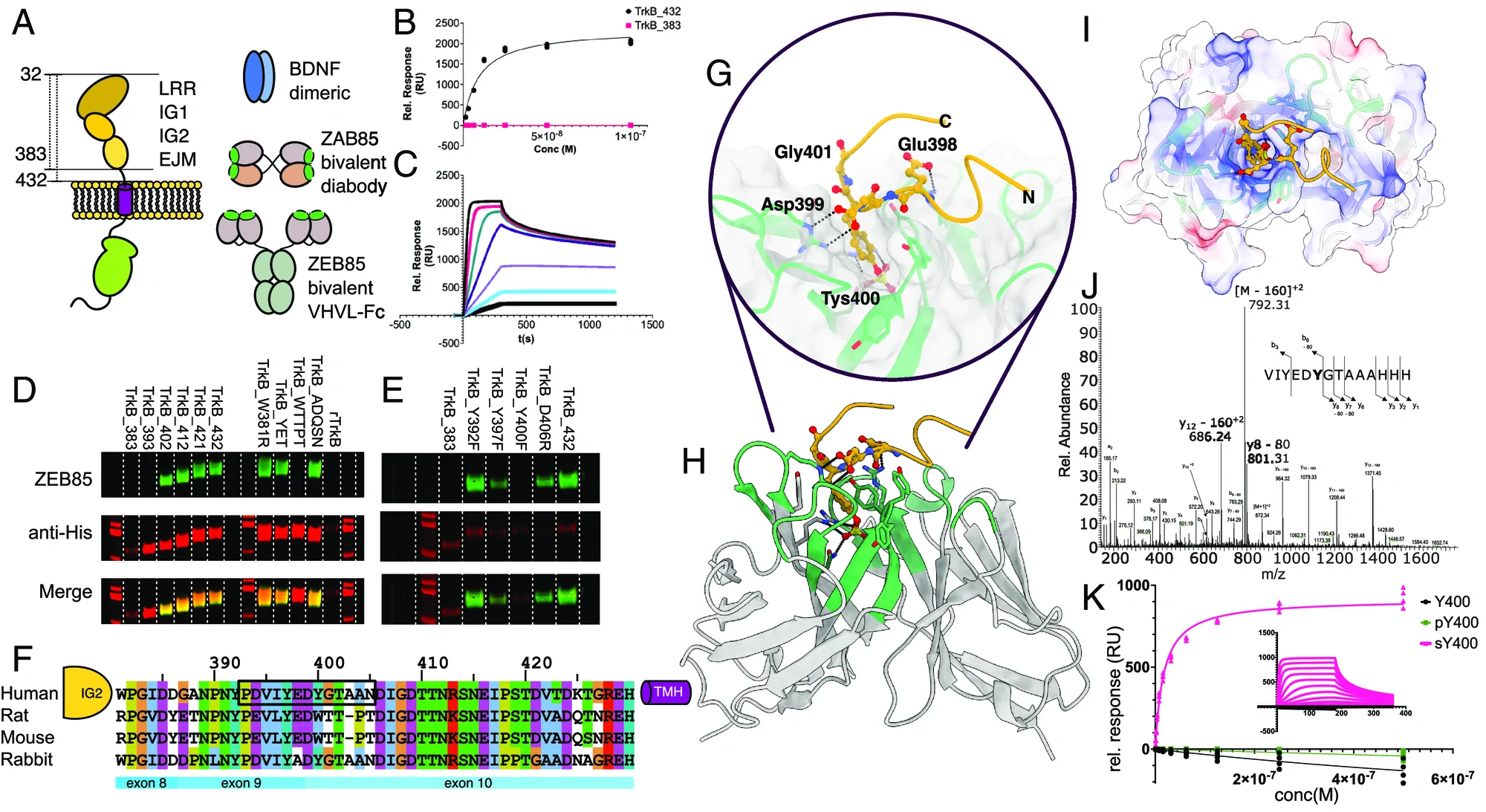
The full agonist ZEB85 recognizes a sulfotyrosine motif in the TrkB extracellular juxtamembrane domain. (*A*) Schematics of molecules described in this study (*B* and *C*) ZEB85 binds to TrkB^432^ but not TrkB^383^ with high affinity (K_D_ = 0.24 nM ± 0.08 nM (n = 7)). (*D*) Representative western blots probing with ZEB85 (green) or anti-His tag control (red) showing that the ZEB85 epitope can be localized to residues 393 to 405 of TrkB, with (*E*) Tyr^400^ being a critical determinant. (*F*) comparison of selected TrkB orthologue eJM sequences between IG2 domain and the transmembrane helix (TMH). (*G* and *H*) Structure of ZEB85 V_H_V_L_ bound to a motif in the TrkB eJM epitope showing a deep pocket for the sulfotyrosine moiety formed by the CDRs and (*I*) the close electrostatic complementarity (*J*) Mass spectrometry evidence for sulfotyrosine at Tyr^400^ for recombinant human TrkB ectodomain digested with bromelain. (An enlarged version is provided as *SI Appendix*, Fig. S7). (*K*) sTyr^400^-modified peptide reconstitutes nanomolar binding affinity to ZEB85 [K_D_ = 0.52 nM ± 0.71 nM (n = 4)], while unmodified (black) and phosphotyrosine-modified (green) peptides shows no detectable binding.

To understand the molecular basis as to how ZEB85 mimics BDNF activation of TrkB, we first sought to map the ZEB85 epitope using purified proteins containing either the full human TrkB extracellular module (residues 32 to 432, TrkB^432^) or lacking the eJM (residues 32 to 383, TrkB^383^) (Fig. 1*A*) We used surface plasmon resonance (SPR) to measure binding of ZEB85 and found that ZEB85 bound TrkB^432^ with nanomolar affinity (K_D_ = 0.24 nM ± 0.08 nM), but showed no detectable binding to TrkB^383^ implicating the TrkB eJM as a harboring a key binding epitope (Fig. 1 *B* and *C*). As the eJM is predicted to be unstructured, we used a western blotting approach to refine the epitope more precisely. We generated a series of finer TrkB truncations and transiently transfected constructs into HEK293 cells for analysis of their conditioned media for ZEB85 binding (Fig. 1*D*). We observed a dramatic loss of ZEB85 binding between the TrkB^402^ and TrkB^393^ truncations. We then examined human/rat chimeras in the eJM, taking advantage of the observation that ZEB85 activates human TrkB but is much less potent toward rodent TrkB (15). We identified four regions of substantial sequence divergence within the eJM and swapped these regions from the rat sequence into human TrkB. This generated human/rat chimera constructs TrkB_W381R, TrkB_YET, TrkB_WTTPT, and TrkB_ADQSN used to produce corresponding recombinant proteins (Fig. 1*D*). Only TrkB_WTTPT, corresponding to residues 400 to 405, showed no binding to ZEB85. Thus, both TrkB truncations and human/rat chimera experiments converged independently on residues 393 to 405 of TrkB as the key binding determinant of ZEB85 (Fig. 1*F*). Surprisingly a synthetic peptide that included this region (residues 383 to 432 of TrkB) bound only weakly to ZEB85 with micromolar affinity (K_D_ 2.34 µM ± 0.91 µM), 4 orders of magnitude less than observed for recombinant TrkB^432^ (*SI Appendix*, Table S2).

We reasoned that the discrepancy between ZEB85 affinity for the synthetic peptide and recombinant TrkB^432^ may be due to a posttranslational modification in this region, and therefore explored that possibility by mass spectrometry. We found that our Expi293-expressed TrkB had a substoichiometric +80 Da modification at residue Tyr^400^ (Fig. 1*J* and *SI Appendix*, Fig. S7), which could correspond to either a phospho- or sulfotyrosine modification. We were unable to distinguish the ~0.0095 Da between sulfate (+79.9568 Da) or phosphate (+79.9663 Da) modification, but noted there was no evidence of an ammonium ion at 216.04, the presence of which would indicate a phospho modification (28). We prepared a panel of Y to F mutants within the ZEB85 epitope and showed that a Y400F mutation rendered the interaction between ZEB85 and TrkB undetectable, implicating Tyr^400^ modification as the critical determinant (Fig. 1*E*). Comparison of the amino acid sequence of TrkB surrounding the sulfotyrosine site (YEDYGTA) with other known sulfotyrosine-containing human proteins (*SI Appendix*, Fig. S1) revealed that the acidic sequences flanking Tyr^400^ are similar to those found in known sTyr-containing proteins such as Nidogen-1 (DEDsYDLA) and Complement C4 (sYEDsYEsYD) (27). This suggested that this TrkB tyrosine side chain could be a suitable substrate for tyrosine-O-sulfotransferases known to reside in the Golgi (25, 26). The residues flanking the sTyr did not fit a motif in extracellular proteins phosphorylated by (for example) the secreted kinase VLK that are modified at a G-pY-P motif (29). Based on this, we considered the sulfotyrosine modification as more likely. We then used SPR to measure the affinity of ZEB85 for TrkB peptides with an unmodified Tyr^400^, a phosphotyrosine 400, or sulfotyrosine 400. The sulfopeptide bound robustly to ZEB85 (0.52 nM ± 0.71 nM), recapitulating the tight binding measured for the recombinant TrkB^432^, whereas the unmodified peptide and phosphotyrosine-containing peptide showed no detectable binding (Fig. 1*K* and *SI Appendix*, Table S2). Differences in formal charge between sulfo- and phospho-groups may account for this finding which unequivocally confirms that TrkB bears a sulfotyrosine modification at position 400 necessary for ZEB85 recognition.

### ZEB85 Recognizes a Linear sTyr-Motif within the TrkB Extracellular Juxtamembrane Region.

To confirm the central role of sTyr^400^ and identify whether flanking residues contribute to binding ZEB85, we determined crystal structures of a single chain Fv region of ZEB85 (“ZAB85”) either with or without the sTyr^400^-containing synthetic peptide (Acetyl-PDVIYEDsYGTAAN-NH_2_). Data processing and refinement statistics can be found in *SI Appendix*, Table S1. The connecting linker between the V_L_ and V_H_ domains is disordered, but further refinement and biophysical analysis revealed that ZAB85 is a diabody with domain-swapped V_L_ and V_H_ domains (30) (*SI Appendix*, Fig. S2). The apo structure revealed a deep cleft composed of aromatic and basic sidechains from CDRH1 & 3, with a non-CDR Arg^76^ and Asn^61^ sidechain from CDRH1 at the base of the pocket. In this apo structure, this pocket is partially occupied by a molecule of HEPES (from the protein buffer) with the sulfate-moiety pointing deep into the cleft.

The ZAB85-sTyr peptide complex structure showed unambiguous electron density for the core interacting region of the sTyr^400^-modified peptide. The structure reveals how the sTyr^400^ sidechain penetrates deep into the CDRH1/CDRH3 pocket (Fig. 1 *G* and *H*), with extensive electrostatic interactions between the aforementioned non-CDR Arg^76^ and CDRH1 Asn^61^ residues at the base of the pocket and the sulfate moiety. This binding pocket contains a single positive charge to bind to the sulfo-moiety, thus explaining the selectivity between sulfo- and phosphotyrosine containing peptides. Hydrophobic interactions are evident between the aromatic group of sTyr^400^ and three tyrosine side chains present in CDRH3. Furthermore two salt bridges between Asp^399^ of TrkB and Arg^80^ (CDRH2), and of Glu^398^, and Arg^241^ (CDRL3) (Fig. 1*I*) contribute to the interaction between TrkB and ZEB85.

Each of the two copies of the TrkB/ZAB85 complex in the asymmetric unit exhibit a positive phi mainchain torsion angle at TrkB Gly^401^ indicating a key structural role for a glycine residue at this position. Amino-terminal to Glu^398^ in the TrkB peptide is a less well-ordered hydrophobic motif (Val-Ile-Tyr). Tyr^397^ forms a hydrophobic interaction with Tyr^128^ (CDRH3), the sidechain of Ile^396^ approaches the Tyr^182^ sidechain, and the sidechain of Val^395^ approaches Tyr^181^. The two copies in the asymmetric unit diverge in this region, suggesting they are less important for the binding of ZEB85. We conclude that the full TrkB epitope recognized by ZEB85 corresponds to Y-E-D-sY-G (TrkB^397-401^).

**Fig. 2.**
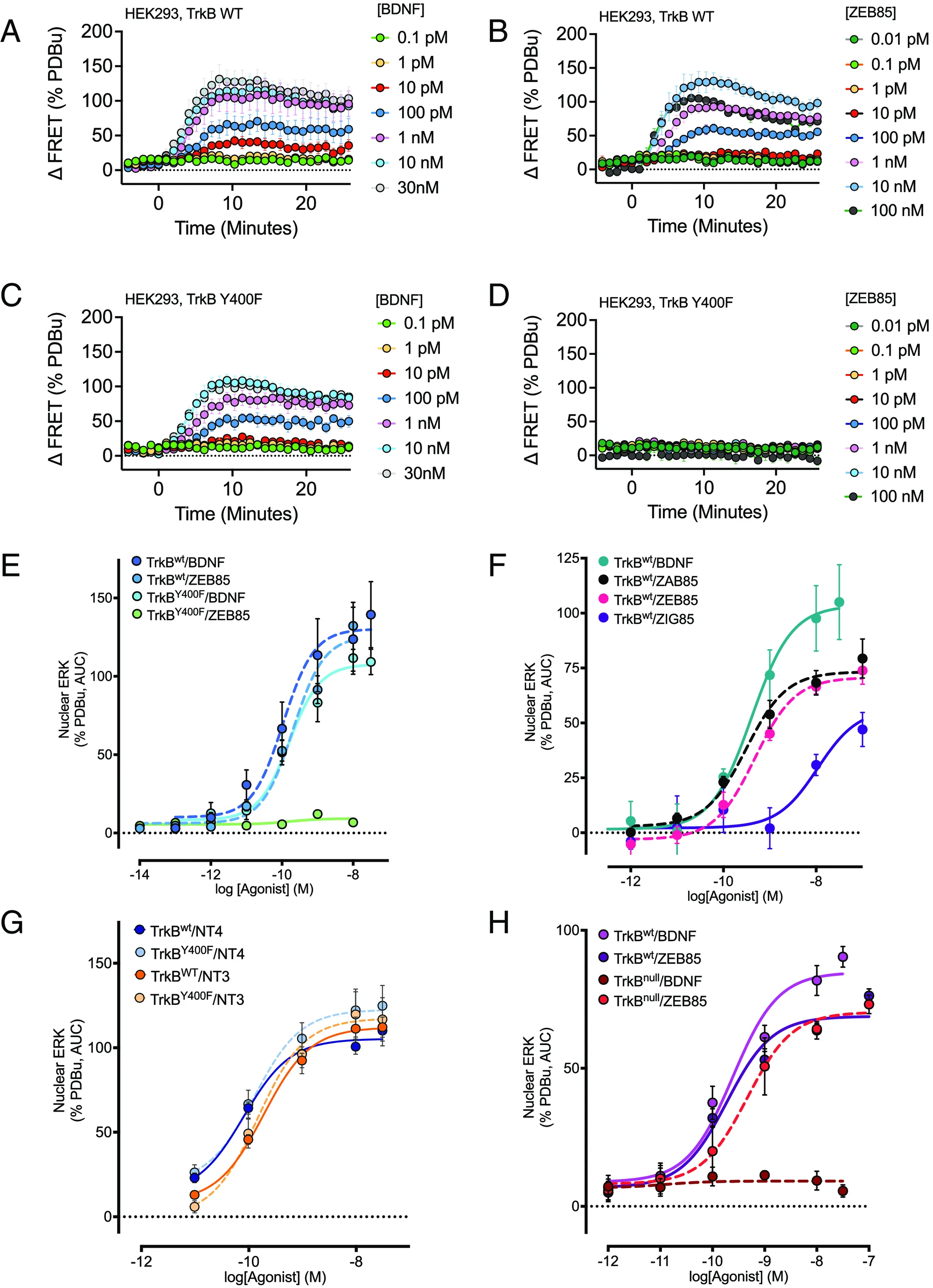
Cellular evidence that the Y400 sulfation site is crucial for ZEB85 agonist activation of TrkB signaling. (*A*) BDNF and (*B*) ZEB85 robustly activate wild-type TrkB signaling in transfected HEK293 cells using a nucEKAR FRET sensor measuring nuclear accumulation of phospho-ERK. (*C*) BDNF fully activates TrkB Y400F signaling, but ZEB85 does not (*D*). (*E*) Comparison of concentration–response curves from panels (*A*–*D*) for ZEB85 or BDNF stimulation of wild type or Y400F TrkB signaling. (*F*) Comparison of different ZEB85 formats as a Fab (ZAB85) or full Ig antibody (ZIG85) showing presentation of bivalent TrkB paratopes impacts agonism efficiency. (*G*) Y400F mutant does not significantly impact TrkB ERK signaling in response to NT3 or NT4 stimulation. (*H*) Comparison of EC50 curves from examining activation of WT TrkB, or a TrkB^null^ (D298R/H299R/M379R) mutant that is deficient in BDNF binding but still responds to ZEB85. Data from 4 to 5 independent replicates with triplicate wells. Mean ± SEM.

To validate our structural findings *in cellulo*, we assessed the effect of the Y400F substitution on human TrkB signaling in HEK293 cells. We measured TrkB activation by cotransfecting a genetically encoded FRET-based sensor of ERK activity to track the real-time accumulation of nuclear phospho-ERK (pERK) downstream of TrkB (31). Both BDNF and ZEB85 activated wild-type TrkB even at low concentrations, with EC_50_ values of close to 0.2 nM for each ligand [pEC_50_ BDNF = 9.82 ± 0.26 (n = 5), pEC_50_ ZEB85 9.715 ± 0.16 (n = 5)] (Fig. 2 *A* and *B*). However, only BDNF was able to activate the TrkB Y400F variant (Fig. 2*C*), while no ERK signaling was observed when TrkB Y400F was challenged with ZEB85 (Fig. 2 *E* and *D*). This demonstrates the exquisite sensitivity of ZEB85 for sY-modified TrkB. We conclude that the sulfation of Tyr^400^ is an absolute requirement for activation of human TrkB by ZEB85 but not for BDNF. Given our observation that ZAB85 formed a diabody, we tested whether the ZEB85 Fc domain fused to its scFv domain was dispensable, testing ZAB85 in the same real-time ERK assay with TrkB. ZAB85 exhibited a comparable EC_50_ to ZEB85 indicating the Fc domain was not crucial (Fig. 2*F*). However, when ZEB85 is reformatted as a full two-chain IgG antibody (ZIG85), its potency drops by several orders of magnitude (pEC_50_ ZIG85 7.37 ± 0.24 (n = 4); Fig. 2*F* and *SI Appendix*, Fig. S3), suggesting that the relative spacing of the sY binding sites is key to efficient TrkB activation.

### Design of a BDNF-Insensitive TrkB from the Structure of a TrkB^383^–BDNF Complex.

**Fig. 3.**
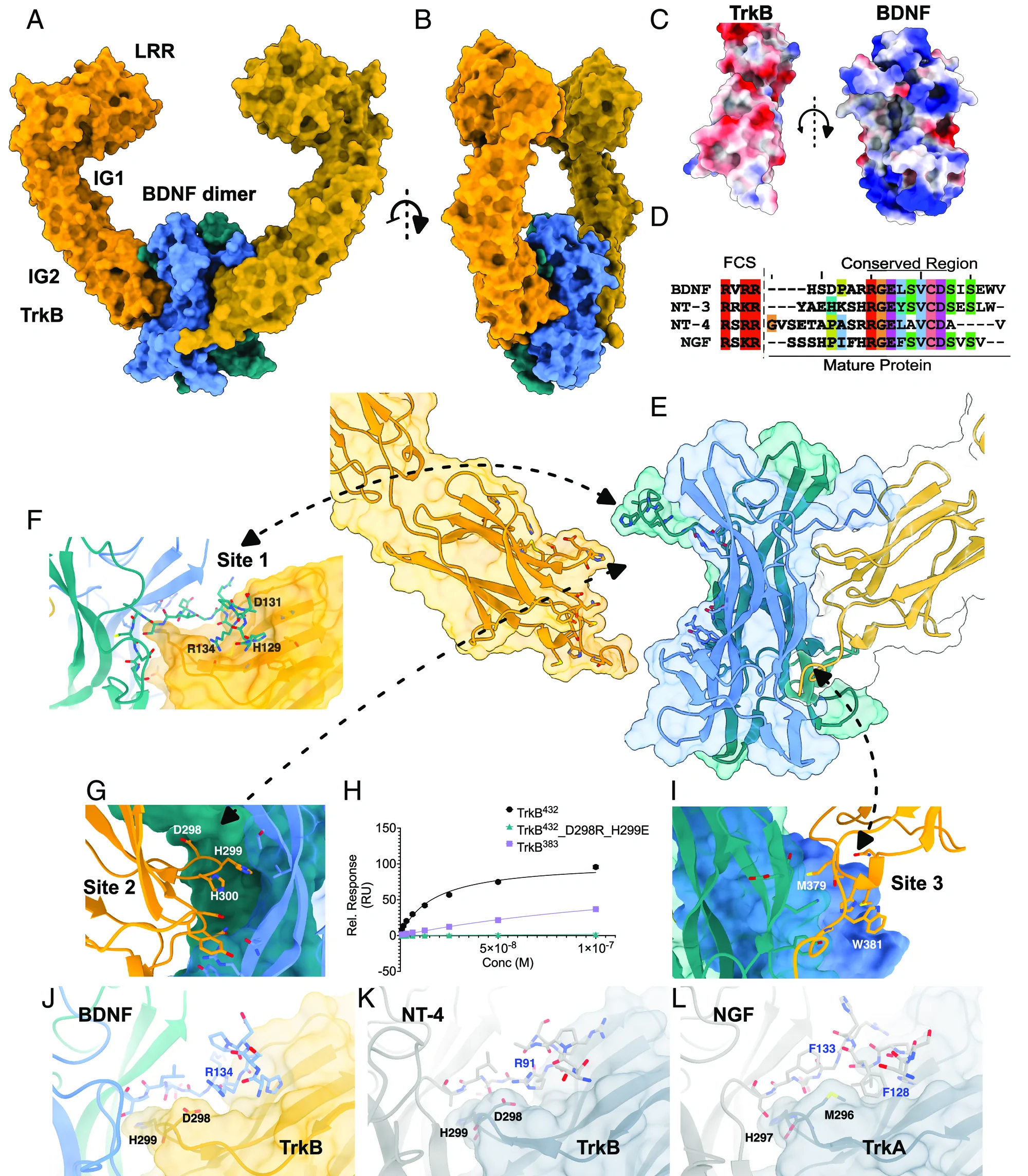
Structure of the human TrkB-BDNF complex and comparison of binding surfaces with other neurotrophins. (*A* and *B*) Orthogonal views of the crystal structure of TrkB-BDNF complex with two main interaction sites. (*C*) TrkB-BDNF interaction depicted in an “open book” view, revealing electrostatic complementarity. (*D*) Comparison of mature neurotrophic factor amino terminal sequences following the furin cleavage site (FCS). (*E*) Separated views for TrkB and BDNF interaction revealing shape complementarity for the three major contact sites. (*F*) The mature amino terminus of BDNF makes contacts with one face of TrkB IG2 (site 1). (*G*) Loops from IG2 of TrkB at the “waist” of BDNF (site 2), making extensive contacts to both BDNF protomers. (*H*) SPR binding data show that TrkB^32-432^ binds BDNF more tightly than TrkB^32-383^, [TrkB^432^ K_D_ = 9.8 nM ± 6.24 nM (n = 3), TrkB^383^ K_D_ = 35.7 nM ± 18.4 nM (n = 3)] and that charge substitution mutants in one loop (D298R/H299E) abolish in vitro binding of BDNF to TrkB ectodomain. (*I*) Site 3 showing the packing of Met^379^ against a BDNF protomer (*J* and *K*) Close-up view highlighting the roles of TrkB Asp^298^ or TrkA Met^296^ interacting with the Site 1/amino terminal extensions of BDNF (*J*, this study) NT-4 (*K*, PDB 1HCF) and NGF (*L*, PDB 2IFG).

The reported competition of ZEB85 and BDNF for TrkB binding (15) appeared at odds with the quite distinct molecular determinants predicted for BDNF within IG2 of TrkB and shown here for ZEB85 in the TrkB eJM. Therefore, we sought to define a structure of BDNF bound to TrkB. We also wanted to use this structure to guide the design of a TrkB mutant unable to respond to BDNF (that has yet to be reported). We prepared a complex using purified recombinant human TrkB^383^ and BDNF (residues 129 to 247) and determined its crystal structure (Fig. 3 *A* and *B*). The diffraction data were anisotropic but gave good quality density maps and a well-refined structure was obtained with good stereochemistry (*SI Appendix*, Table S1).

The overall structure of the TrkB^383^- BDNF 2:2 complex is similar to that of TrkA^ECM^-NGF structure (16), with the dimeric BDNF ligand sandwiched between the 2nd IG domains of the two TrkB ectodomains. There is a hinge motion between IG domains 1 and 2 of TrkA and TrkB that accounts for most of the gross structural differences between the two structures. BDNF forms a compact homodimer with a buried surface area of 1,120 Å^2^ per monomer. The buried surface area between BDNF and one TrkB monomer is 1,040 Å^2^, and the interfaces show extensive shape and electrostatic complementarity (Fig. 3 *C* and *E*). The interface between TrkB and BDNF can be roughly segmented into three regions: an amino-terminal site (site 1) where the mature N terminus from one molecule of BDNF packs against one face of TrkB IG2 (Fig. 3*F*) and a second site (site 2) where residues from several loops (including residues 298 to 300) from IG2 pack against a shared interface composed of both BDNF protomers in the dimer (Fig. 3*G*), and a minor hydrophobic site (site 3) at the membrane-proximal end of IG2 where Met^379^ interacts with a shallow hydrophobic pocket on one BDNF protomer (Fig. 3*I*).

For site 1, the entire mature N terminus of BDNF from His^129^ to Ile^138^ is ordered, revealing a key region that differs substantially from that of NGF (32), both in terms of sequence and structure when bound to their canonical Trk receptors (Fig. 3 *D* and *F*). The first residue of mature BDNF after pro-BDNF processing is His^129^ and both its sidechain and mainchain pack into a shallow groove on the face of TrkB IG2 formed by residues Phe^291^, Pro^304^, Thr^306^, His^343^, and the Cys^302^-Cys^345^ disulfide bond. We note that residues BDNF^129-135^ were disordered in heterodimeric structures of BDNF determined in the absence of TrkB receptor (33, 34), suggesting that these amino acids become ordered on receptor binding. Comparison of our TrkB-BDNF structure with the TrkA-NGF structure suggests that site 1 confers specificity, with the amino-terminus of NGF/BDNF and 298 to 300 (TrkB numbering) loop of the receptor sterically hindering binding with mismatched ligands, supporting a hypothesis first put forward by Pattarawarpan and Burgess (35). We also note that BDNF used in this study is the only commercial source with a native amino-terminal histidine, rather than an inauthentic initiator methionine or vector-derived leader sequence as used in many other studies (36).

To validate these contacts and to design a TrkB variant insensitive to BDNF, we generated a panel of structure-guided mutations in TrkB probing residues at the interface with BDNF and assessed their affinities for BDNF by SPR and expression level (*SI Appendix*, Fig. S6). While several mutants had a major impact on BDNF interaction, a double charge-reversal D298R/H299E mutant in the 290-loop of TrkB Ig2 domain showed no detectable interaction with BDNF in vitro (Fig. 3*H*). This mutant was then tested in the HEK293 nuclear ERK cotransfection assay, which unexpectedly retained activity. We therefore combined an additional mutation to give a triple mutant D298R/H299E/M379R (hereafter, TrkB^null^) that was completely unresponsive to BDNF in cellulo and gave no nuclear ERK signal, but retained full activity for ZEB85 activation (Fig. 2*H* and *SI Appendix*, Fig. S4). We rationalized that the D298R/H299E mutant disrupted binding to TrkB extracellular domain but not membrane-localised full-length TrkB signaling because the latter exhibits a higher affinity for BDNF than the soluble TrkB ectodomain, perhaps through presentation of a preformed dimer mediated by interaction between the transmembrane helices as described by Kot *et al*. (37). However the triple D298R/H299E/M379R mutation was sufficient to render TrkB unresponsive to BDNF in cells. This allows TrkB to be engineered to selectively respond to either BDNF or to ZEB85 through quite different mutations.

We then tested whether other neurotrophins known to activate TrkB (NT-3 and NT-4) might be differentially impacted by either of our Y400F and TrkB^null^ mutants. We again used the cotransfected HEK293 ERK activity FRET sensor and found that TrkB signaling by NT-3 and NT-4 did not differ significantly between WT and Y400F variants but was abolished in the TrkB^null^ triple mutant as found for BDNF (Fig. 2*G* and *SI Appendix*, Fig. S5). Therefore, we conclude that the sulfotyrosine modification on TrkB does not influence neurotrophic factor signaling through TrkB, but that NT-3, NT-4, and BDNF cannot signal through our TrkB^null^ mutant.

We compared our BDNF-TrkB structure reported here to equivalent neurotrophin-Trk structures deposited in the PDB: the structure of TrkA bound to NGF [PDB:2IFG, (16)] and the structure of the TrkB IG2 domain complexed with NT-4 [PDB:1HCF, (17)]. When comparing all three binding surfaces, we observe that the TrkB His^299^ (TrkA His^297^) in site 2 is conserved both in sequence and in orientation for both Trk receptors. Conversely TrkB Asp^298^, which forms an electrostatic interaction with BDNF Arg^134^/NT-4 Arg^91^ (numbered Arg^11^ in PDB entry 1HCF) is not conserved in TrkA. In TrkA Met^296^ makes hydrophobic interactions with residues NGF Phe^128^ and Phe^133^ (Phe^7^ and Phe^12^ in PDB entry 2IFG) within the N-terminal tail of mature NGF. Indeed, TrkA Met^296^ would sterically clash with BDNF Arg^134^ and NT-4 Arg^91^, impeding their interaction and further supporting the hypothesis that it is the interaction of site 2 with the amino-terminal tail of the neurotrophins (at site 1) that confers specificity for the different Trk receptors (Fig. 3 *J*–*L*). In TrkC, the equivalent residue to TrkB Asp^298^ would be TrkC Arg^316^, thus each human Trk receptor isoform differs substantially at this residue position consistent with neurotrophin selectivity and affinity being controlled through the neurotrophin amino terminal residues. In contrast, site 3 (TrkB & TrkA Met^379^) is conserved among Trk receptors, as are the neurotrophin residues it interacts with; primarily BDNF Trp^147^, NT-4 Trp^103^ (Trp^23^ in PDB entry 1HCF), and NGF Trp^142^ (Trp^21^ in PDB entry 2IFG).

### BDNF Binding Surface With TrkB Extends Into the Extracellular Juxtamembrane.

**Fig. 4.**
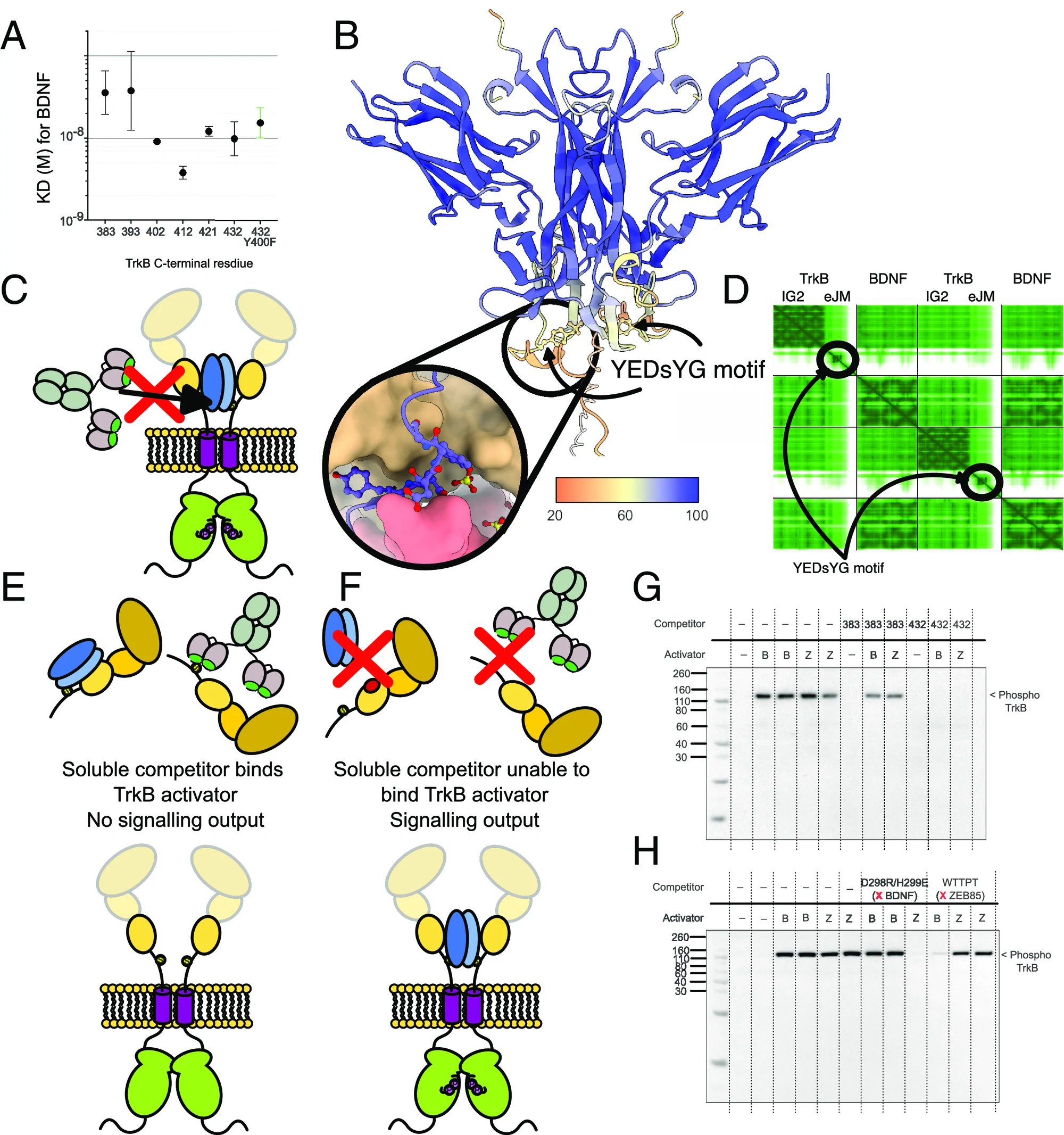
Overlap of BDNF and ZEB85 binding sites at the extracellular juxtamembrane region of TrkB. (*A*) Truncation series of the TrkB eJM reduces the affinity of TrkB for BDNF with a sharp K_D_ jump between TrkB^32-393^ and TrkB^32-402^ (n = 3) (*B*) AlphaFold3 model for BDNF and TrkB coloured by Predicted Local Distance Difference Test (pLDDT) reveal an extended TrkB/BDNF interface with good electrostatic complementarity in which residues 383 to 402 of the eJM interact with and tuck under the membrane proximal surface of BDNF. (*C*) In this AF3 model, the ZEB85 epitope is sterically occluded by BDNF binding, explaining the observed competition. (*D*) The predicted aligned error (PAE) plot. (*E* and *F*) Competition assay in H9 neurons between soluble recombinant TrkB ectodomain variants and endogenous full-length TrkB. (*G*) TrkB^32-383^ lacking the eJM cannot compete with full-length TrkB for either ZEB85 or BDNF leading to full-length TrkB activation. TrkB^32-432^ with the eJM motif sequesters both TrkB and BDNF preventing full-length TrkB activation. (*H*) A TrkB mutant selectively deficient in either BDNF (D298R/H299E) or ZEB85 (sulfotyrosine motif deficient) binding cannot sequester the corresponding ligand and cannot block full-length TrkB activation.

Our results revealed a clear distinction between ZEB85 and BDNF requirements for stimulation of TrkB with a sTyr^400^ modification. However, it was still possible that other elements in the eJM could be required for BDNF binding even if sTyr^400^ was not critical. We therefore investigated whether the initial series of TrkB truncations used to identify the ZEB85 epitope showed any impact on BDNF interaction by SPR. Results indicated a marked fall off in binding affinity for TrkB constructs ending between residue 393 and 402 (Fig. 4*A* and *SI Appendix*, Table S2). TrkB truncations ending at or before residue 393 had apparent K_D_ values ~40 nM, while truncated proteins ending after residue 402 showed apparent K_D_ values ~10 nM. This suggested that an eJM binding contact for BDNF existed beyond the TrkB^383^ boundary used for structure determination. Additionally, we were unable to detect any significant difference between TrkB^432^ WT (K_D_ = 9.8 nM ± 6.24 nM) and TrkB^432^Y400F (K_D_ = 15.3 nM ± 6.6 nM) binding to BDNF, consistent with the nuclear ERK FRET sensor experiments.

Attempts to crystallize a TrkB^402^–BDNF complex (with a longer eJM encompassing the sTyr modification) were not successful. We therefore used AlphaFold3 (AF3) (38) to predict a potential binding site for BDNF to include the eJM (Fig. 4 *B* and *D*). This gave a plausible model in good agreement with the SPR data, suggesting an extended interaction surface that continues as far as residue 405. Residues in this region have a predominantly acidic nature and therefore have good charge complementarity with the highly basic BDNF. Residues beyond this were not predicted to make contacts to BDNF, had low confidence pLDDT scores, and exhibited the characteristic “barbed-wire” appearance of a nonpredictive AlphaFold result in this region (39).

To validate the interaction of TrkB eJM with BDNF in a cellular context, we used a soluble TrkB ectodomain competition assay using mature human neurons derived from H9 embryonic stem cells that expressed endogenous full-length TrkB (15). The assay probed the ability of different soluble TrkB variants to sequester either BDNF or ZEB85 ligand and thereby block endogenous full-length TrkB activation in neurons as detected by tyrosine autophosphorylation (Fig. 4 *E* and *F*). For this assay, we first used two soluble variants of TrkB extracellular domain to compete for BDNF or ZEB85 binding and prevent activation of cellular full-length TrkB. Whereas TrkB^432^ could sequester both BDNF and ZEB85 as predicted, preventing full-length TrkB activation (Fig. 4*G*), TrkB^383^ was unable to sequester either ZEB85 or BDNF despite the presence of the IG2 domain. For ZEB85, this can be explained by the loss of the sTyr^400^-motif, whereas the inability of TrkB^383^ to sequester BDNF agrees with the SPR data demonstrating that the eJM is required for BDNF interaction in this competition assay. To probe this further, we used the same competition assay to examine the D298R/H299E charge reversal mutant that abolished BDNF binding to purified TrkB ectodomain in vitro and a human/rat chimera that removed the sY-motif of human TrkB (labelled WTTPT, Fig. 1*D*) disrupting ZEB85 binding. Here there was a clear separation of function; BDNF was unable to bind the soluble TrkB D298R/H299E mutant resulting in no competition with full-length TrkB and thus resulting in neuronal TrkB activation. In contrast, ZEB85 was captured by this mutant blocking full-length TrkB activation. Conversely, TrkB WTTPT could compete with BDNF blocking full-length TrkB activation, whereas the same mutant could not bind ZEB85 and therefore was unable to block full-length TrkB activation (Fig. 4*H*).

As our AF3 TrkB-BDNF model also includes the ZEB85 epitope as part of an extended BDNF binding site, this explains the competition experiments in neurons and the observed displacement of ZEB85 from TrkB-expressing HEK293 cells by BDNF detected by FACS sorting (15). From these combined results, we conclude that the TrkB eJM contributes binding determinants for both BDNF and ZEB85 interaction, but only ZEB85 has an absolute requirement for the sulfotyrosine motif (Fig. 4*C*).

## Discussion

The discovery of biological agonists against cell surface receptors has seen a recent renaissance, providing a source of safe, developable, and readily manufactured therapeutics (40). The advent of clinically approved solutions for antibody biological delivery to the CNS presents a significant opportunity for developing neurology-focused therapeutics against key molecular targets (41). As well as their potential transformational impact on neurological disease, antibody agonists also provide highly specific tools for discovery science including providing a route to elucidate their mechanism of action (40). TrkB agonist antibodies have long been recognized as promising tools to selectively activate TrkB (reviewed in ref. 42, 43). Several potent antibodies have been described and are being pursued as potential therapeutics in neurodegeneration including 29D7, AB2 and AB20, mAb1D7, ZEB85, MM12, and AS86 (13–15, 44–46). We have focused on ZEB85 as a potent agonist that closely mimics BDNF in its TrkB-mediated signaling and transcriptional responses (15). Here, we identify a short linear sulfotyrosine motif within human TrkB as the epitope critical for activation by ZEB85. We also show that mutation of this site (Y400F) in a cellular context abolishes ZEB85-mediated signaling in transfected HEK293 cells, implicating this modification in cellular responses to ZEB85. The response of TrkB to ZEB85 observed in H9-derived neurons (15) and sensitivity to removal of the sY-motif, suggests the presence of the sulfotyrosine modification in native TrkB in a neuronal context. Using structure-guided separation of function mutations, we also show that ZEB85 and BDNF have overlapping TrkB binding sites within the extracellular juxtamembrane region. Despite the challenges preparing high-quality diffracting crystals of TrkB:BDNF complex (see supplementary methods, *SI Appendix*, Table S1) we were able to identify the residues in the binding interface, and validated those observations by mutation in both in-vitro binding assays (*SI Appendix*, Fig. S5), with cellular activity assays (Fig. 2*H* and *SI Appendix*, Fig. S4) and from structural homology to the higher-resolution structure of TrkB IG2 NT-4 complex [PDB entry 1HCF, (17)]. Also, we note that the B-factor/Atomic displacement parameter (ADP) distribution within the BDNF-TrkB structure is lowest at the BDNF–TrkB interface region, allowing confident assignment of contacts in this region.

### Overlap of BDNF and ZEB85 Binding Site at the TrkB Extracellular Juxtamembrane Region.

In spite of their overall sequence similarities, the three neurotrophin receptors TrkA, TrkB, and TrkC show very different functional behaviors in vivo, including the induction of cell death from TrkA and TrkC, but not TrkB, in the absence of their respective ligands (47). The four mammalian neurotrophin ligands BDNF, NT4, NGF, and NT3 distinguish the Trk receptors with varying degrees of selectivity. The extracellular juxtamembrane region of TrkB is a major source of sequence divergence among the Trk receptors, where the eJM segments of TrkA and TrkC are markedly shorter than TrkB. Our finding that BDNF and ZEB85 binding sites overlap at the eJM is supported by competition experiments (15) but had not been previously demonstrated (Fig. 4). A closer analysis of the eJM region by directly measuring BDNF affinity constants and using a competition assay for soluble TrkB ECM ligand sequestration, point to this region being necessary for BDNF binding, in addition to the previously reported TrkB IG2 domain. This explains how the nanomolar ligand ZEB85 could be competed off TrkB by BDNF, through the latter’s picomolar TrkB affinity, by engaging the eJM segment. An important implication of this finding is that it extends the TrkB/BDNF binding site beyond the IG2 domain and toward the membrane. Such an extended interface had been originally proposed by Ibáñez and colleagues in a study detailing the generation of a pan-neurotrophin-1 (PNT-1) that could activate TrkA, TrkB, and TrkC (48). Even a single chimeric molecule using an NT-3 backbone and BDNF variable region V (motif Ser^222^-Lys^223^-Lys^224^-Arg^225^-Ile^226^-Gly^227^) was able to enhance TrkB interaction and increased the survival of nodose neurons (48). In the crystal structure presented here, this basic region V motif in BDNF is fully accessible and predicted by AlphaFold to engage the acidic eJM of TrkB, consistent with our findings. One limitation of this study is the affinities are measured using soluble TrkB proteins rather than on the membrane. However, the competition assay does directly compare the ability of full-length TrkB to compete with soluble forms. Equally, using a FRET-based sensor to indirectly measure TrkB activation also confirms our in vitro findings. Both approaches could be used to identify directly the BDNF residues contacting TrkB eJM. Reported findings for other TrkB agonists such as MM12 have implicated binding determinants within the eJM but have not mapped the site to a specific motif such as the sulfotyrosine motif reported here (45). It is possible some of the reported TrkB agonist antibodies may also target sTyr^400^ and with the tools reported here these can now be readily tested. Other neurotrophins may also utilize their canonical Trk receptor eJM region. For example, a recent report for the NGF^painless^ mutant R100W proposes a TrkA eJM acidic contact site is impacted by this mutation, perturbing PLCγ signaling and uncoupling nociception signaling (49). The importance of the eJM in other RTKs has also been demonstrated, with different splice variants of ErbB4 and FGFR2 varying in their eJMs, which in turn leads to different signaling outputs (50) or ligand binding (51) respectively.

**Fig. 5.**
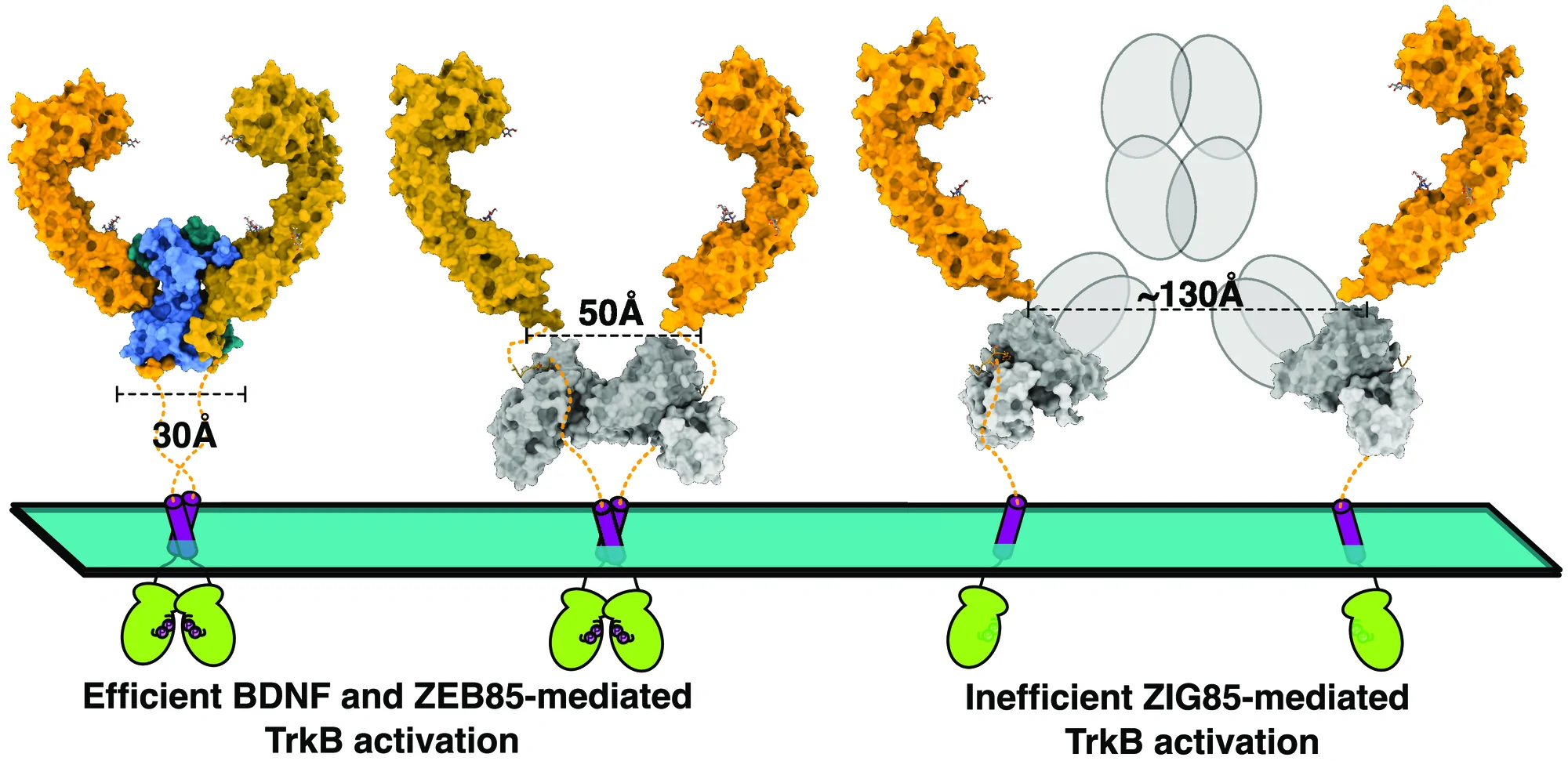
Schematic model of observed geometric constraints on TrkB activation. BDNF (blue) and ZEB85/ZAB85 (silver) promote close association of TrkB protomers (orange) (~30 to 50 Å separation of TrkB protomer eJMs), leading to efficient activation. The bulkier ZIG85 can engage two TrkB monomers but holds them further apart (~130 Å) leading to less efficient signaling.

Our results also show that the full antibody format ZIG85 variant, with different paratope spacing leads to a much less efficient TrkB activation (Fig. 2*F*).This finding might have broader implications and suggests that refactoring antibody agonists into scFv-Fc scaffolds and even diabodies be brought into the toolkit of agonist antibody development alongside Fc-(52) and disulfide-engineering (53). Based on our structures, BDNF and ZEB85/ZAB85 have much smaller paratope–paratope distances of ~30 Å (BDNF) and ~50 Å (ZAB85), whereas mean paratope–paratope distances in IgG are ~130 Å ± 40 Å (Fig. 5) (54). Therefore, we conclude that, much like other receptor tyrosine kinases (55), the spacing enforced by the TrkB domains closest to the membrane is of great import when considering efficient signaling output. In the case that TrkB exists in preformed, inactive dimers in an unliganded state (as proposed in ref. 56), the wide spacing of the paratopes in ZIG85 may preclude binding within the same inactive dimer, thus failing to efficiently convert inactive, dimeric TrkB to an active dimeric TrkB.

### A Potential Physiological Role of the Sulfotyrosine Motif Unique to TrkB.

This study identifies a sulfotyrosine modification within the TrkB receptor tyrosine kinase that we have extensively validated by a combination of mass spectrometry, structural biology, biophysical, and cell signaling assays. The extracellular sY-motif shares homology to known TPST substrates and we exclude the possibility of ZEB85 recognizing a similarly sized phosphotyrosine modification. The functional role of this enigmatic modification is not yet clear and may not be easy to explore in commonly used animal models given the absence of this tyrosine residue in the corresponding mouse and rat sequences. Given the affinity of ZEB85 for this modification, we propose the TrkB sulfotyrosine motif could provide a protein interaction platform for physiological ligands, potentially analogous to intracellular SH2 domains for phospho-tyrosine (57). TrkB sulfotyrosine partners are likely in neuronal contexts that could functionally impact TrkB signaling, stability, or trafficking. Precedents exist for extracellular protein sulfotyrosine modification contributing to protein ligand–receptor interactions. For example, complement C5a binding to the C5a receptor is enhanced by three orders of magnitude by the presence of a pair of sulfotyrosine residues (58). Equally, multiple chemokine receptors are known to be sulfated at tyrosine sites within their amino-termini to influence the affinity of cognate chemokine interactions (59). The functional role of the motif is likely specific to mammalian TrkB since the motif is conserved in all major mammalian families apart from rodent TrkB sequences, which responds only weakly to ZEB85. Also, the motif is unique to TrkB, as TrkA and TrkC lack any tyrosines in their respective eJM regions, preventing exposure to such a modification during Trk receptor maturation and passage through the trans-Golgi network. It is known that the sulfotyrosine modification can be substoichiometric and it is possible that variation of the frequency of modification would allow for fine-tuning of sulfotyrosine-dependent TrkB functions, as is seen for CCR5 (60). For TrkB, the sulfotyrosine stoichiometry is sufficiently high to elicit a full biological response to ZEB85 in all neuronal lines tested. For TrkB, there are a number of reported binding partners for the TrkB extracellular domain that might potentially exploit this motif, such as SLITRK5 (61) or the mGlu2 (62) and mGlu5 receptors (63, 64). However, further work will be required to investigate if there are physiological roles and partners for the sTyr modification of TrkB.

Overall, our data point to a clear role that the TrkB eJM plays in receptor recognition by both BDNF and ZEB85. While ZEB85 requires a previously undetected and acid-labile sulfotyrosine modification in this region, neurotrophins BDNF, NT3, and NT4 that can bind TrkB, do not require sulfotyrosine. This report highlights how studying antibody agonists can reveal previously uncharacterized features and motifs relevant to endogenous ligand recognition. Our findings point to new hypotheses and biology to explore for sulfotyrosine-modified TrkB, such as its interactome and intracellular trafficking. Our study also highlights the increasing need in this postgenomic and post-AlphaFold era to detect, characterize, and understand the contribution of underexplored posttranslational modifications in human health and disease (65).

## Materials and Methods

Please see *SI Appendix*, *Extended Materials and Methods* for more information

### Protein Production and Purification.

All proteins in this study were expressed in and secreted from Expi293 or Expi293 GntI- cells (Gibco) growing in Freestyle293 media (Gibco), transiently transfected using a PEI-based method. Mature BDNF was a generous gift from Professor Mart Ustav (Icosagen, Estonia).

### Surface Plasmon Resonance.

All surface plasmon resonance experiments were performed on a Biacore S200, in HEPES- or Tris-buffered saline with 0.005% (v/v) P-20 surfactant. Chip chemistry, ligand, and analyte are detailed in *SI Appendix*, Table S2.

### Crystallization, Data Collection, and Structure Determination.

Purified ZAB85 (5 mg/mL) was crystallized by sitting-drop vapor-diffusion at 293 K. Crystals were obtained in 17% PEG3350, 0.24 M CaCl2, 0.1 M BisTrisPropane, pH 6.8, cryocooled for data collection using 20% ethylene glycol as a cryoprotectant. ZAB85–peptide complex used ZAB85 (5 mg/mL) mixed with ten-fold molar excess of solid sY400-peptide was dissolved in the protein solution prior to crystallization by sitting-drop vapor-diffusion at 293 K. Crystals were grown in 20% PEG8k, 0.2 M MgCl2, 0.1 M Tris pH 8.5, and were then cryocooled for data collection using 20% ethylene glycol as a cryoprotectant. TrkB 1-383 (8 mg/mL) was mixed with human BDNF to give 1:1 molar ratio for crystallization. Crystals were obtained in 30% PEG4k/glycerol mix, 0.1 M Bicine/Tris buffer at pH 8.5 and 0.12 M Morpheus ethylene glycol mix. Diffraction data were collected at beamlines I04 (ZAB85 and TrkB 1-383-BDNF) or I24 (ZAB85-sY-peptide) at the Diamond Light Source. Diffraction images were indexed, integrated, and scaled using DIALS (66). Phases were estimated using Phaser (67) followed by model building in Coot (68) and refinement using Phenix (69) or Refmac (70). Final data processing and model statistics are given in *SI Appendix*, Table S1. All structural figures were generated using ChimeraX (71). Further experimental details are provided in *SI Appendix*.

### TrkB Signaling FRET Sensor Assay for Nuclear-ERK Activity.

We used a genetically encoded, FRET-based sensor of ERK activity (the extracellular signal-regulated kinase activity reporter, EKAR) to assess TrkB signaling in transfected HEK293 cells (31).

### Competition Assay Using iPSC-Derived H9 Neurons Expressing Endogenous Full-Length TrkB.

Neurons differentiated from human ES cells (H9 clone) were prepared as described in (72).

## Supplementary Material

Appendix 01 (PDF)

## Data Availability

Structure data have been deposited in PDB (30PP, 30PU, and 30PO) (73–75). All study data are included in the article and/or *SI Appendix*.
