# Supplementary material for: A sulfotyrosine motif in the human TrkB extracellular domain is required for activation by the agonist antibody ZEB85
Source: Proc Natl Acad Sci U S A. 2026 Oct 29;123(44):e2618780123. doi: 10.1073/pnas.2618780123 (PMC13732090; doi:10.1073/pnas.2618780123)
Supplement: Supplementary file 1 [file pnas.2618780123.sapp.pdf]

PNAS

Supporting Information for:

A sulfotyrosine motif in the human TrkB extracellular domain is required for activation by the agonist antibody ZEB85

David C. Briggs[a], Ryan T. Duffy[b], Sarah Ateaque[c], Sarah Maslen[d], Hema Nagaraj[e], Yves-Alain Barde[c], Peter S. DiStefano[f], Ronald M. Lindsay[f], Paul C. Armstrong[g], Chloe J. Peach[b], Neil Q. McDonald[a,1]

[a] – Signalling and Structural Biology Lab, The Francis Crick Institute, London NW1 1AT, United Kingdom

[b] – Centre of Membrane Proteins and Receptors (COMPARE), School of Life Sciences, University of Nottingham, Nottingham NG7 2UH, United Kingdom.

[c] – School of Biosciences, Cardiff University Cardiff, CF10 3AX, United Kingdom

[d] – Proteomics Science Technology Platform, The Francis Crick Institute, London NW1 1AT, United Kingdom

[e] – Chemical Biology Science Technology Platform, The Francis Crick Institute, London NW1 1AT, United Kingdom

[f] – Zebra Biologics Inc, 1014 Old Marlboro Road, Concord, Massachusetts 01742, USA

[g] – Blizard Institute, The Faculty of Medicine and Dentistry, Queen Mary University of London, The Blizard Building, 4 Newark Street, London E1 2AT, United Kingdom.

[h] – Institute of Structural and Molecular Biology, School of Natural Sciences, Birkbeck College, Malet Street, London, WC1E 7HX, United Kingdom

[1] To whom correspondence may be addressed. Email: neil.mcdonald@crick.ac.uk

This PDF file includes:

Supporting text
Extended Materials and Methods
Figures S1 to S7
Tables S1 to S2
SI References

Supporting Information Text

ZAB85 is a diabody in solution and in the crystal lattice.

To readily crystallize the ZEB85 V_HV_L region containing the TrkB epitope, we removed the Fc region from the expression construct. We anticipated that removal of the dimeric Fc region would produce a monovalent scFv (ZAB85). However, we found that ZAB85 was as potent as the bivalent ZEB85 in the FRET sensor assay for TrkB signalling. We investigated the monomeric/dimeric status of ZAB85 using mass photometry, which unambiguously revealed an in-solution molecular weight of double the expected monomeric molecular weight, consistent with a dimer even at low concentrations (Fig. S2*A*). Inspection of the crystal packing (indexed and scaled in point group 6/*mmm*) indicated that the disordered linker between the V_H V_L domains was unlikely to connect adjacent V_H V_L domains, so we considered a domain swapped 'diabody' arrangement (1) (Fig. S2*B*). Reprocessing the X-ray data in space group P1 (removing any imposition of symmetry), reduced the overall resolution of the processed data, but showed more electron density for the disordered linker allowing us to extend the linker model (Fig. S2*C*). This unambiguously demonstrated that ZAB85 is a diabody (2). Diabodies form when the V_H V_L domain connecting linker is sufficiently short to force a non-covalent dimerization with another V_H V_L molecule, creating a stable diabody with two antigen-binding sites. We determined two slightly different diabody structures in the asymmetric unit (Fig. S2*E,F* "Diabody AB" and "Diabody MN"), consistent with the two independent copies in the 6/*mmm* asymmetric unit. Further evidence for the dimeric state of ZAB85 in-solution came from size exclusion chromatography-coupled small angle X-ray scattering (SAXS) experiments providing data that are most consistent with the "AB" diabody (Fig. S2*D,G*) from the crystal lattice. Based on the amino acid sequences of the ZEB85 and ZIG85 constructs, we conclude that the V_HV_L region of ZEB85 also adopts the same diabody configuration (hence the comparable TrkB activation profile (Fig. 2*F*), whereas in the full-antibody format of ZIG85, the extra domains and separate light and heavy chains adopts a classical IgG configuration with a much wider separation (134 Å ± 40 Å for IgG (3), compared with ~50 Å for ZEB/ZAB85) of the CDR regions in the two arms of the antibody.

Extended Materials and Methods

Protein production and purification

All proteins in this study were expressed in and secreted from Expi293 or Expi293 GntI- cells (Gibco) growing in Freestyle293 media (Gibco), transiently transfected using a PEI-based method previously reported (4). A codon optimised human TrkB construct 1-383 (including native signal sequence 1-31) was synthesised by Geneart. This construct contained sequence changes to remove sites of non-conserved N-linked glycans to make the recombinant protein more amenable to structural study by X-ray crystallography. The construct was subcloned into pcDNA3.1 vector with a carboxy-terminal hexahistidine-tag (6xHis-tag) to facilitate purification and detection. The construct for the full ECD of TrkB (1-432) was created by inserting the native sequence for TrkB residues 384-432 into this vector using Gibson assembly, retaining the 6xHis-tag. Glycan site mutations were introduced using Q5-polymerase site directed mutagenesis protocol (New England Biolabs). All TrkB extracellular proteins were harvested from the conditioned media with Ni-NTA Sepharose (Qiagen) washed with Tris-buffered saline (TBS) containing 10 mM imidazole and eluted using TBS with 500 mM imidazole.

ZEB85 (V_HV_L-FC) was similarly generated by Geneart and subcloned into pcDNA3.1 and harvested using protein G Sepharose, and a low pH elution (0.2 M Glycine pH 3.5) step which was then immediately neutralised using 1 M Tris pH 9.0. A construct for the smaller ZAB85 (V_HV_L-3C-StrepII) was created using

Gibson assembly, by inserting the coding sequence for the secretion sequence and V_HV_L region into a pcDNA3.1 vector containing a C-terminal 3C cleavage site and a single StrepII-tag for purification. ZAB85 was harvested from conditioned media using StrepTactin-Sepharose (IBA Lifesciences), washed with TBS, and then cleaved from the resin using GST-3C protease overnight at 4ºC. The GST-3C was then removed from the eluate using Glutathione Sepharose 4B resin(Cytiva). All proteins were subjected to a final size exclusion chromatography polishing step using a Superdex 200 Increase column (Cytiva). This material was then aliquoted and snap frozen in liquid nitrogen and stored at -70ºC until needed. Mature BDNF was a generous gift from Professor Mart Ustav (Icosagen, Estonia).

Surface plasmon resonance

All surface plasmon resonance (SPR) experiments were performed on a Biacore S200, in HEPES or TBS with 0.005 %(v/v) P-20 surfactant. Chip chemistry, ligand, and analyte are detailed in Table S2. To obtain quantitative data for the BDNF - TrkB interaction, we found that if TrkB ECD proteins were the immobilised ligand and BDNF used as the analyte, the SPR sensorgrams were uninterpretable due to the approximately zero off rate consistent with the reported picomolar affinity of this interaction (5, 6). However, by immobilising BDNF, we found the apparent K_D for TrkB ECD proteins was reduced to ~nM (presumably to steric hinderance or non-optimal binding-site presentation on the sensor chip) giving an off-rate within the capabilities of the instrument. This allowing us to compare BDNF binding kinetics for different TrkB variants.

Western blotting to determine ZEB85 epitope

All gels were run using BOLT gels (Life Technologies) in a MES running buffer. Transfer to nitrocellulose membranes was done using an iBlot2 or iBlot3 semi-dry transfer device. A blocking buffer of TBS + 0.1 % Tween (TBS-T) + 1 % BSA + 5 % milk powder was used for all primary antibodies. Incubation with primary antibodies was done overnight at 4ºC. The membranes were then washed 3x10minutes with TBS-T before incubation with an appropriate LiCor secondary antibodies in fluorescence blocking buffer (Antibodies online) for 1 hour at room temperature. The membranes were then washed 3x10min with TBS-T again before visualising the using a LICor Odyssey Scanner (LICORbio).

Detection of sulfotyrosine by mass spectrometry.

Purified $TrkB^{32\text{-}402}$ was reduced and alkylated in solution and digested with bromelain at 1:20 ratio. The resulting peptides were analysed by nano-scale capillary LC-MS/MS using an Ultimate U3000 HPLC (ThermoScientific Dionex, San Jose, USA) to deliver a flow of approximately 300 nL/min. A C18 Acclaim PepMap100 5 µm, 100 µm x 20 mm nanoViper (ThermoScientific Dionex, San Jose, USA), trapped the peptides prior to separation on a C18 Acclaim PepMap100 3 µm, 75 µm x 250 mm nanoViper (ThermoScientific Dionex, San Jose, USA). Peptides were eluted with a 60 minute gradient of acetonitrile (2 % to 50 %). The analytical column outlet was directly interfaced via a nano-flow electrospray ionisation source, with a hybrid quadrupole orbitrap mass spectrometer (Q-Exactive Orbitrap, ThermoScientific, San Jose, USA). Data dependent analysis was carried out, using a resolution of 70,000 for the full MS spectrum, followed by ten MS/MS spectra. MS spectra were collected over a m/z range of 350–1800. MS/MS scans were collected using a threshold energy of 27 for higher energy collisional dissociation (HCD). LC-MS/MS data were searched against an in-house protein database using the Mascot search engine programme (Matrix Science, UK)(7). Database search parameters were set with a precursor tolerance of 10 ppm and a fragment ion mass tolerance of 0.15 Da. No enzyme was chosen for bromelain and variable modifications for oxidized methionine, carbamidomethyl cysteine, and sulphated serine, threonine and tyrosine. MS/MS data were validated using the Scaffold programme (Proteome Software Inc., USA)(8). All data were additionally interrogated manually.

Synthesis and purification of sY containing peptides

The neopentyl-protected tyrosine-sulfated peptides were synthesised on a 0.1mmole scale, using solid-phase peptide synthesis on a ResPep Automated Peptide Synthesiser (CEM) using 5 equivalents of N(a)-Fmoc amino acids and 4.9 equivalents of HATU (Hexafluorophosphate Azabenzotriazole Tetramethyl Uronium) as the coupling reagent and 10 equivalents of Diisopropylethylamine (DIPEA). The Fmoc deprotections were carried out on the synthesiser using 20% piperidine in DMF (Dimethylformamide) containing 1% formic acid. Standard Fmoc protected amino acids were used, along with Fmoc-Tyr(S03nP)-OH (CAS 878408-63-0). All amino acid were double coupled.

Following chain assembly, peptides were cleaved from the resin and protecting groups removed by addition of a 10ml cleavage cocktail (95% TFA (trifluoracetic acid), 2.5% H2O, 2.5% TIS (triisopropylsilane) and 2.5% EDT (ethanedithiol) if the peptide contains cysteine or methionine. After 4h, the resin was removed by filtration, and the peptides were co-precipitated with 40mls of cold diethyl ether on ice. The peptides were isolated by centrifugation, then dissolved in 10mls H_2O and freeze dried overnight. Following lyophilisation, the neopentyl (nP) group was removed by dissolving the peptide in a minimal amount of DMSO (40mg in 300ul). The solution was diluted to 1.5ml with 2M NH_4OAc and incubated at 37 °C for 6 h. The reaction was monitored by using LC-MS.

After deprotection, the peptides were purified in 40mg batches on a C8 reverse phase HPLC column (Agilent PrepHT Zorbax 300SB-C8, 21.2x250 mm, 7 m) using a linear solvent gradient of 0-20% B over 40mins at a flow rate of 8 mL/min. A binary solvent system [A: H2O /0.08% TFA/1% MeCN (acetonitrile) and B: MeCN /0.08% TFA] was used. The peak fraction was analysed by LC–MS on an Agilent 1290 LC-MSD (Agilent Poroshell 120 EC-C18 2.7um, 3.0x30mm column), using a linear gradient of 5-60% B over 8.5 min at a flow rate of 0.425 mL/min.

Crystal structure determination of ZAB85 alone and bound to TrkB$^{393-405}$ eJM peptide

For ligand-free structure determination, ZAB85 was gel-filtered in HEPES-buffered saline and then diluted 1:3 with milliQ water and concentrated to 5 mg/ml. Crystals grew in 17 % PEG3350, 0.24 M $CaCl_2$, 0.1 M BisTrisPropane, pH 6.8, and were then cryocooled for data collection using 20 % ethylene glycol as a cryoprotectant. Data were collected to 1.88 Å resolution at beamline I04, Diamond Light Source. Diffraction images were indexed, integrated and scaled using DIALS (9) software. Phases were estimated using Phaser (10) and a molecular replacement search model of the VHL portion of 5YAX (11), with the CDR residues removed to reduce the influence of phase bias in the resulting maps. CDRs were readily traced and built into the resulting maps using Coot (12).

For structure determination of ZAB85 bound to a synthetic TrkB$^{393-405}$ peptide containing the sTyr^{400} modification, the ZAB85 was gel filtered in Tris-buffered saline instead of HEPES buffered saline. Again, protein was diluted 1:3 with milliQ water and concentrated to 5 mg/ml. Ten-fold molar excess of solid peptide was dissolved in the protein solution prior to crystal screening. Crystals were grown in 20 % PEG8k, 0.2 M $MgCl_2$, 0.1 M Tris pH 8.5, and were then cryocooled for data collection using 20 % ethylene glycol as a cryoprotectant. Data were collected to 2 Å resolution at beamline I24, Diamond. Diffraction data processing was performed as described for ZAB85 alone. Phases were estimated using Phaser (10) and a molecular replacement search model of the ZAB85 apo form. Final data processing and model statistics are given in Supplementary table 1.

Crystal structure determination of TrkB$^{32\text{-}383}$ bound to mature human BDNF

TrkB 1-383 (32-383 mature protein) was purified from Expi293 GntI- cells to produce homogenous N-linked glycan modifications. This protein was harvested from conditioned media using Ni-NTA Sepharose, and then further purified using size exclusion chromatography in buffer consisting of 20 mM Tris-HCl, pH 8.0, 100 mM Potassium Acetate. The material was then concentrated to 8 mg/ml and mixed with an appropriate amount of human BDNF at 1 mg/ml to yield a 1:1 molar ratio. This material was then used directly for crystal screening. Crystals grew from the Morpheus screen (13) in 30 % PEG4k/glycerol mix, 0.1 M Bicine/Tris buffer at pH 8.5 and 0.12 M Morpheus ethylene glycol mix. These crystals diffracted anisotropically to 3Å - 4.5Å at I04, Diamond Light Source. Data was processed using DIALS (9) – the unit cell parameters were initially identified as C222(1), 57.54 Å x 179.93 Å x 183.81 Å . The data were phased using the TrkA/NGF structure, PDB 2ifg (14), divided into 4 individual search models (TrkA LRR, TrkA IG1, TrkA IG2, NGF) using Phaser (10). Refinement stuck at an R_{Free} of around 40 %, and so we hypothesised that the space group assignment may be incorrect. Zanuda (15) indicated that the true space group was C2. After reprocessing the data in C2 (with β = 90.04º), re-refinement gave a drop in RFree to ~32 %. The data were further processed using STARANISO (16) (Global Phasing) to help ameliorate the effects of the anisotropy. Final data processing and model statistics are given in Supplementary Table 1. All structural figures were generated using ChimeraX (17)

TrkB signalling FRET sensor assay for nuclear-ERK activity.

We used a genetically encoded, FRET-based sensor of ERK activity (the extracellular signal-regulated kinase activity reporter, EKAR) to assess TrkB signalling in transfected HEK293 cells (18). HEK293 cells plated (25,000 cells per well) in black 96-well plates pre-coated with poly-D-lysine were serum-starved following 24h and transfected with nuclear EKAR CFP YFP (40 ng/well; Addgene, #18681) and either FLAG-TrkB (WT) or FLAG-TrkB (Y400F mutation), SnapTag-TrkB (WT) or SnapTag-TrkB^{null} (D298R/H299E/M379R) mutation (20 ng/well) using polyethyleneimine (PEI, 6:1) diluted in PBS solution. Following 48 h, cells were incubated in Hank's Buffered Saline Solution (HBSS)/0.1% Bovine Serum Albumin (BSA) and stimulated with increasing agonist concentrations of either BDNF (0.1 pM-30 nM), ZEB85 (0.01 pM-1000 nM), ZAB85 (1 pM-1000 nM), ZIG85 (1 pM-1000 nM), NT-3 (10 pM-30 nM), or NT-4 (10 pM-30 nM). Phorbol 12,13-dibutyrate (PDBu, 10 µM) was used as a positive control and the buffer solution HBSS/0.1 % BSA was used as a vehicle treatment. Fluorescent emissions (CFP = 480 nm and YFP = 530 nm) were recorded (PHERAstar) over a period 26 minutes, with ligand addition occurring at t = 5 mins. FRET ratio was calculated (530nm/480nm), which provided the "raw data" for analysis. Data was then baseline corrected to the first 5 reads (pre-ligand addition) and then baseline corrected a second time to the vehicle. The data was then normalised to the maximal response of the positive control (PDBu, 100 %) or vehicle (0 %) and expressed as a percentage. Concentration-response data was fit using non-linear regression analysis as log vs. response (three parameters) to determine EC50 values. Data are presented as mean ± SEM and analysed using GraphPad Prism (10.1.2).

Competition assay using iPSC-derived H9 neurons expressing endogenous full-length TrkB

Neurons differentiated from human ES cells (H9 clone) were prepared as described in (19). These neurons were incubated with the appropriate ligand for various lengths of times as indicated. Prior to lysis, the cultures were washed with PBS, and subsequently lysed with 70 µl of RIPA lysis buffer: 50 mM Tris- HCl, pH 7.4, 150 mM NaCl, 1 mM EDTA, 1% Triton-X-100, 0.2% sodium deoxycholate, 0.1% SDS and supplemented with a cocktail of protease and phosphatase inhibitors (Sigma-Aldrich) at 1:100 dilution containing 100 mM 1,10-Phenanthroline, 100 mM 6-aminohexanoic acid, 10 mg/ml aprotinin and 2 mM sodium orthovanadate. Cell lysates were kept on ice for 10 min, then centrifuged for 15 min at 15,000 rpm and the supernatant

transferred to a new tube prior to western blot analysis, or storage at -80°C.4 X NuPAGE loading buffer (containing 0.666 g Tris HCl, 0.682 g Tris Base, 0.8 g LDS (lithium dodecyl sulfate), 0.006 g EDTA (ethylenediaminetetraacetic acid), 4 g glycerol, 0.75 ml SERVA Blue G250 (1 % solution) and 0.25 ml phenol red (1% solution) and 10 X DTT (dithiothreitol) (Sigma-Aldrich) was added to each sample before heating at 70°C for 10 min (in cases where DTT was omitted, samples were heated for 5 min). Samples were then loaded onto a 4-12 % Bis-Tris NuPAGE gel (Invitrogen) and the gel run at 120 V for 1.5 hours. Protein was transferred to nitrocellulose membranes using the wet transfer Mini-Trans Blot Cell (Bio-Rad). The membrane was then washed with PBS and stained with Ponceau Red to allow visualisation of transferred protein. The membrane was then blocked for 1h with blocking solution (5 % western blotting grade blocker (BIO-RAD) and 1 % BSA (Sigma-Aldrich) in 1 X TBS-T) (described below). Following this the blots were probed with rabbit monoclonal anti-P-Trk (Cell Signaling, mAb #4621) diluted 1:4000, goat polyclonal anti-TrkB (R&D Systems) diluted 1:2000, goat polyclonal anti-TrkC (R&D Systems) diluted 1:2000, mouse monoclonal anti-synaptophysin (Sigma-Aldrich S5768) diluted 1:2000 or anti-arc mouse monoclonal (Santa Cruz sc – 17839). The primary antibody was diluted in blocking solution, and incubation was overnight at 4 °C.

The membranes were then washed 3 times with 20 ml TBS-T (10 X TBS (10 X TBS – 25 mM Tris Base, 1.37 mM NaCl, 2.6 mM KCl dissolved in ddH2O. TBS-T: 1 X TBS & 1% Tween-20 (Sigma-Aldrich) for 10 min per wash on a rocker and incubated with the secondary antibody (1:2000) in blocking solution for 1 hour. The primary antibodies were detected with donkey anti-Rabbit HRP-conjugated, anti-goat HRP-conjugated and anti-Mouse HRP-conjugated secondary antibodies (Promega). The membrane was developed using the WesternBright ECL Kit (Advansta). Blots were visualized with the Image Lab software and the Universal Hood III camera system (BIO-RAD). Densitometry analysis of the bands with ImageJ was used to calculate the intensity of the signal for each band. The quality of protein transfer was checked in every case by staining the membranes with Ponceau Red. Normalisation in all cases was deemed acceptable based Ponceau staining as the volume as well as the protein concentrations were found to be consistent between samples (see above). Synaptophysin was therefore not routinely used as a normalisation control.

Small Angle X-ray scattering and mass photometry.

Small angle X-ray scattering (SAXS) data were collected with in-line size exclusion chromatography (SEC-SAXS) at Diamond B21. Briefly, 10 mg/ml ZAB in Tris buffered saline was injected onto a Superdex 200 Increase column and data were collected across the elution volume. Data were analyzed, the buffer background scattering subtracted, and the peak data merged using the ScÅtterIV software(20). Ab-initio envelopes were created using DAMMIN (Dummy Atom Model Minimization) (21). Reciprocal space comparison of raw data and models was generated using CRYSOL (22). Data were plotted using SASPLOT (22). Mass photometry (Refeyn) was carried out using ZAB85 protein at concentrations ranging from 1-5 nM in Tris-buffered saline, according to manufacturer's instructions.

Figures

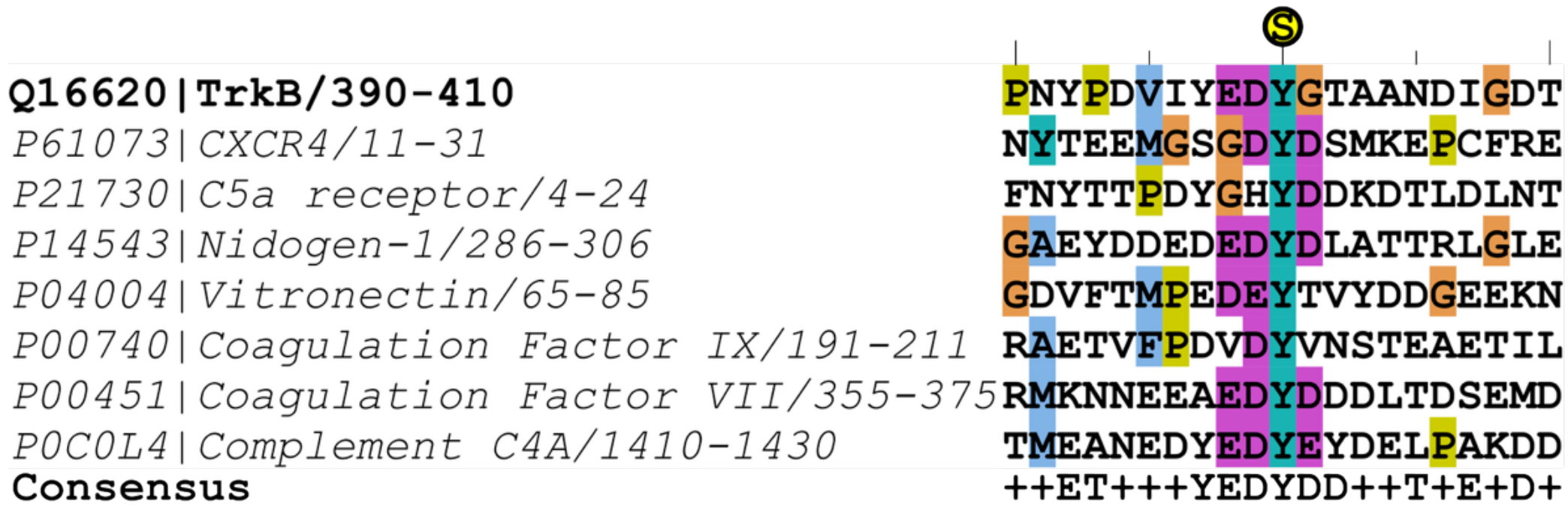

Fig. S1: Similarity of the TrkB sTyr400 motif to known sTyr-containing motifs found in selected human proteins. Uniprot identifiers and sequence extent are indicated.

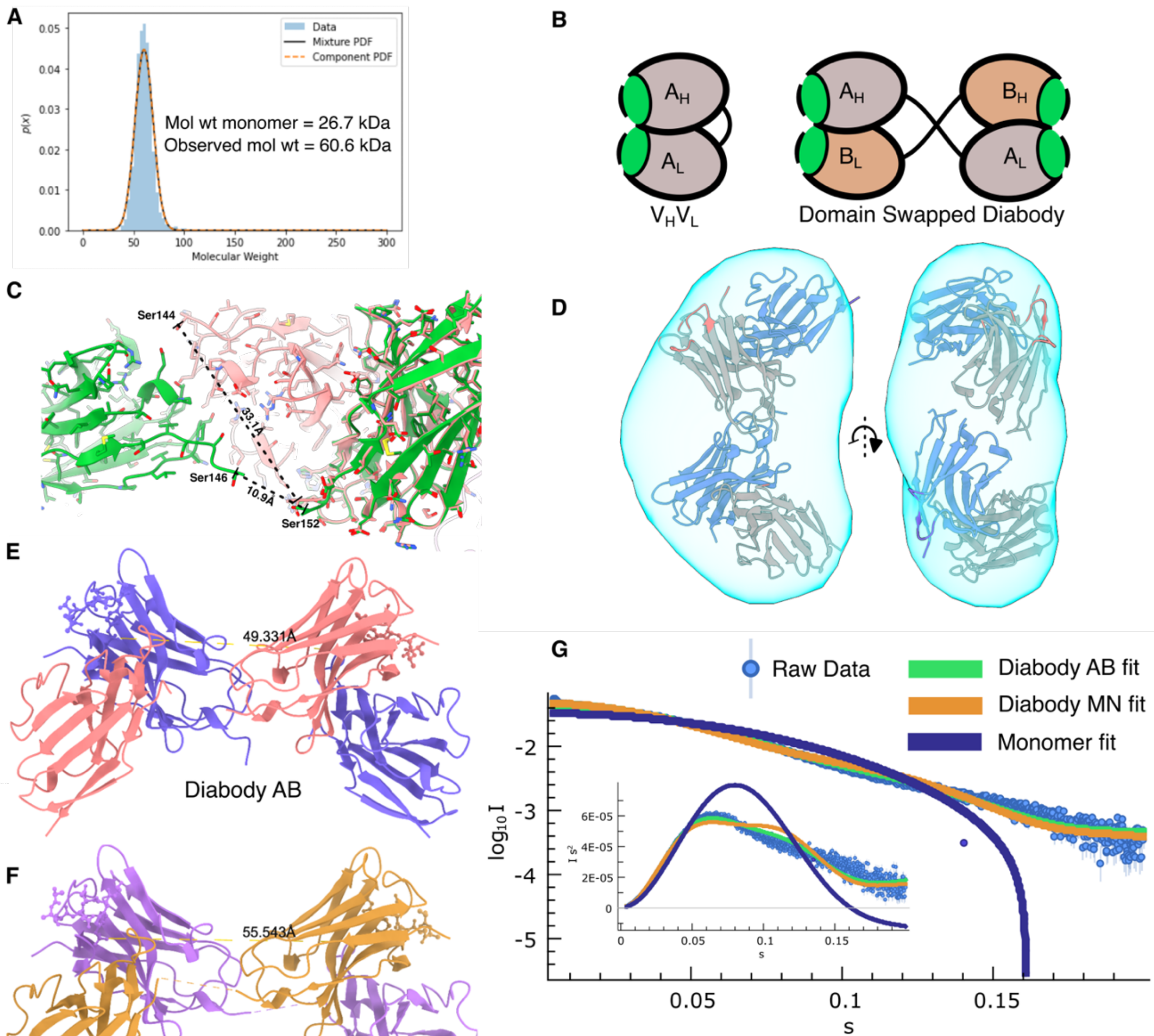

Molecule	Fit to SAXS data (chi2)
Diabody AB	4.28
Diabody MN	12.54
Monomer	197.1

Fig. S2: Crystallographic and in solution evidence that ZAB85 adopts a dimeric state consistent with a diabody. (A) Mass photometry analysis of ZAB85 indicates a dimeric state even at nanomolar concentrations, (B) Schematic of a monomeric compared to a domain-swapped diabody influenced by connecting linker length. (C) The disordered linker distance in the domain swapped, P1 model (green, missing residues 147-151, 10.9 Å) is plausible, whereas the monomeric configuration (pink, missing residues 145-151, 33.1 Å) is not. (D) Ab-initio SAXS DAMMIN envelope is consistent with a diabody configuration. (E,F) the two independent diabodies observed from the P1 cell processed crystal structure. The sTyr-sTyr binding site distances are indicated. (G) Diabody AB is most consistent with the SAXS data, although the Kratky plot (inset) is indicative of some flexibility.

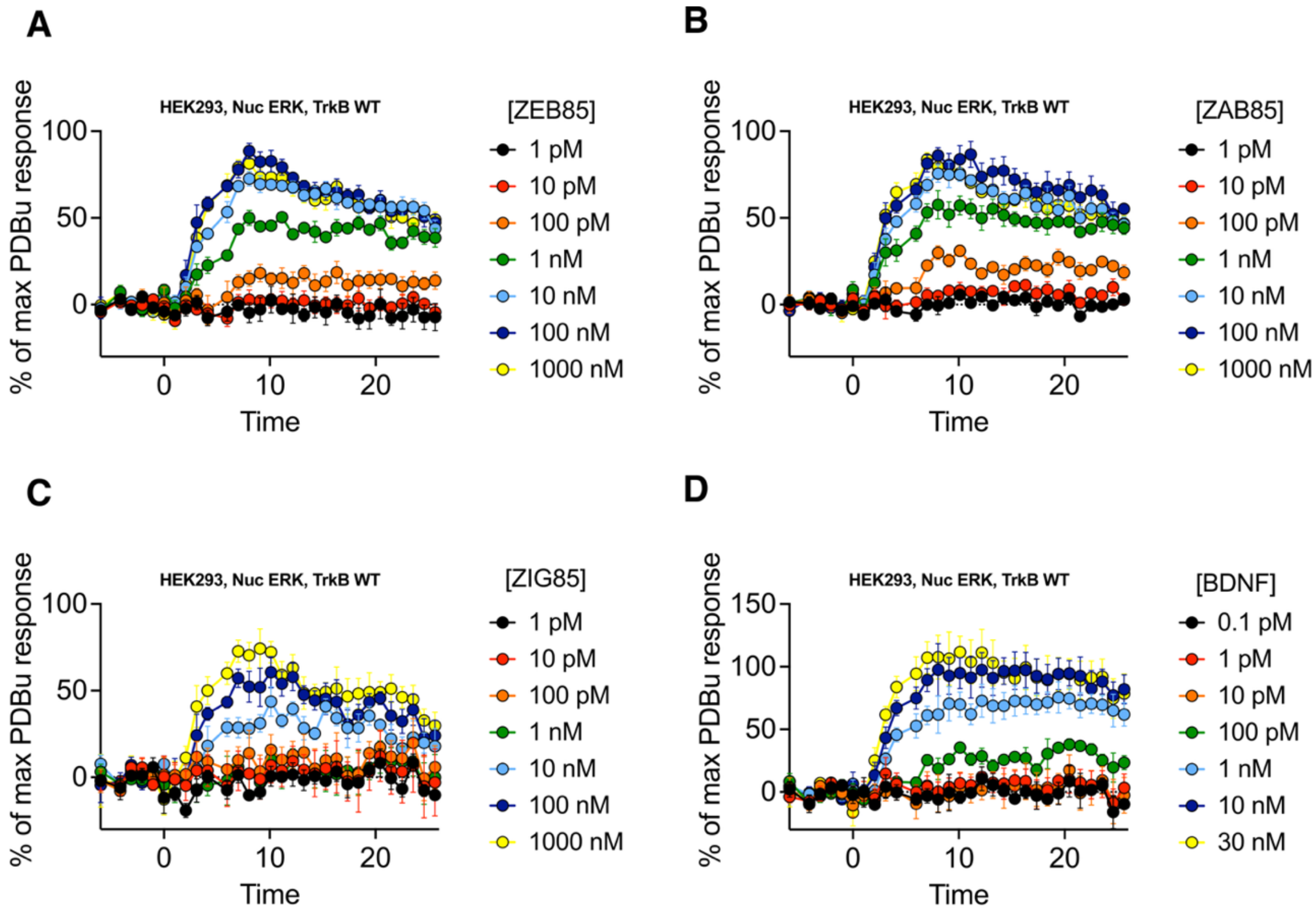

Fig. S3: Real-time ERK signalling in response to ZEB85, ZAB85, ZIG85 or BDNF. HEK293 cells co-transfected with human TrkB and a nuclear ERK FRET sensor plasmids were stimulated with ZEB85 (A), ZAB85 (B), ZIG85 (C) or BDNF (D). Data from 4-5 independent replicates with triplicate wells. Mean ± SEM.

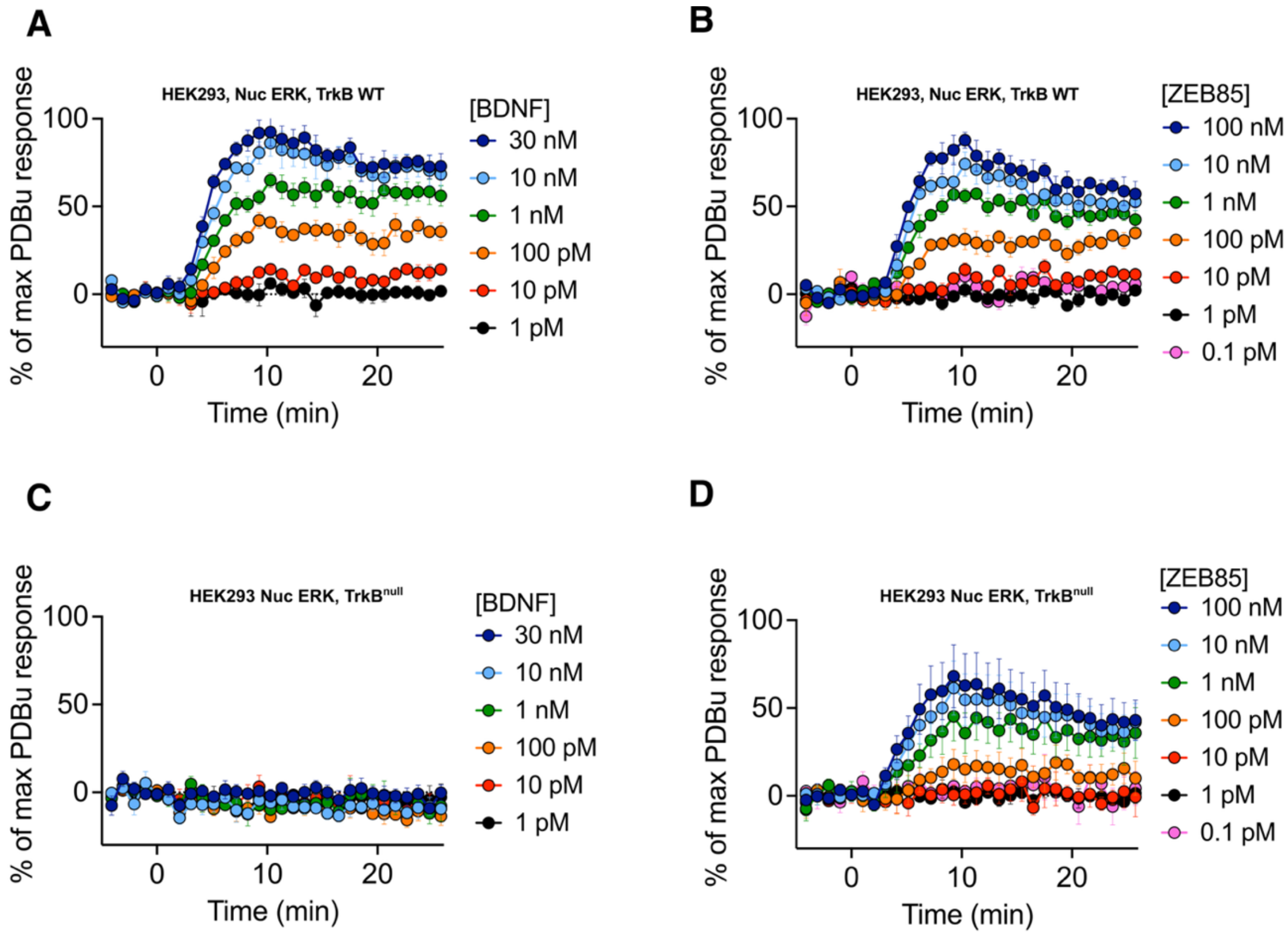

Fig. S4: TrkB^{null} disrupted BDNF-induced ERK signalling, but not ZEB85-induced responses. HEK293 cells co-transfected with mutant forms of human TrkB and a nuclear ERK FRET sensor plasmids were stimulated with ZEB85 or BDNF. Comparison of ERK responses to TrkB WT (A,B) or TrkB^{null} (mutant D298R/H299E/M379R) (C,D) in response to increasing concentrations of BDNF or ZEB85. Data from 4-5 independent replicates with triplicate wells. Mean ± SEM**.**

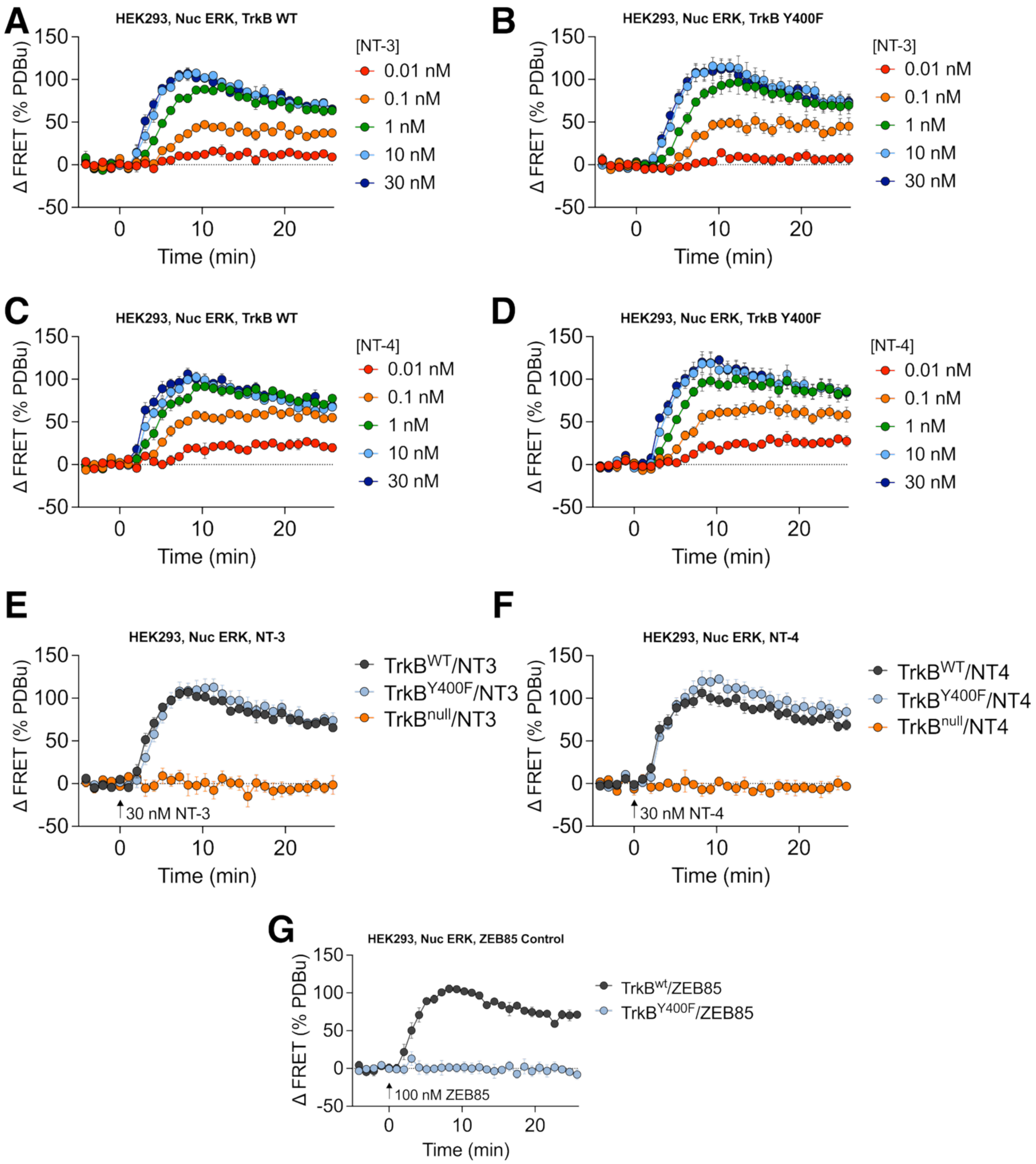

FigS5: Effect of TrkB mutations of NT-3 and NT-4 induced ERK signalling. HEK293 cells co-transfected with mutants of human TrkB and a nuclear ERK FRET sensor plasmids were stimulated with NT-3 or NT-4. TrkB^{Y400F} mutation does not disrupt NT3 (**A,B**) or NT4 (**C,D**) -induced ERK signalling. However, TrkB^{null} (mutant D298R/H299E/M379R) abolished ERK responses to NT3 (**E**) and NT4 (**F**). (**G**) Control experiment confirming the impact of TrkB^{Y400F} on ZEB85 signalling at 100nM ZEB85. Data from 4-5 independent replicates with triplicate wells. Mean ± SEM.

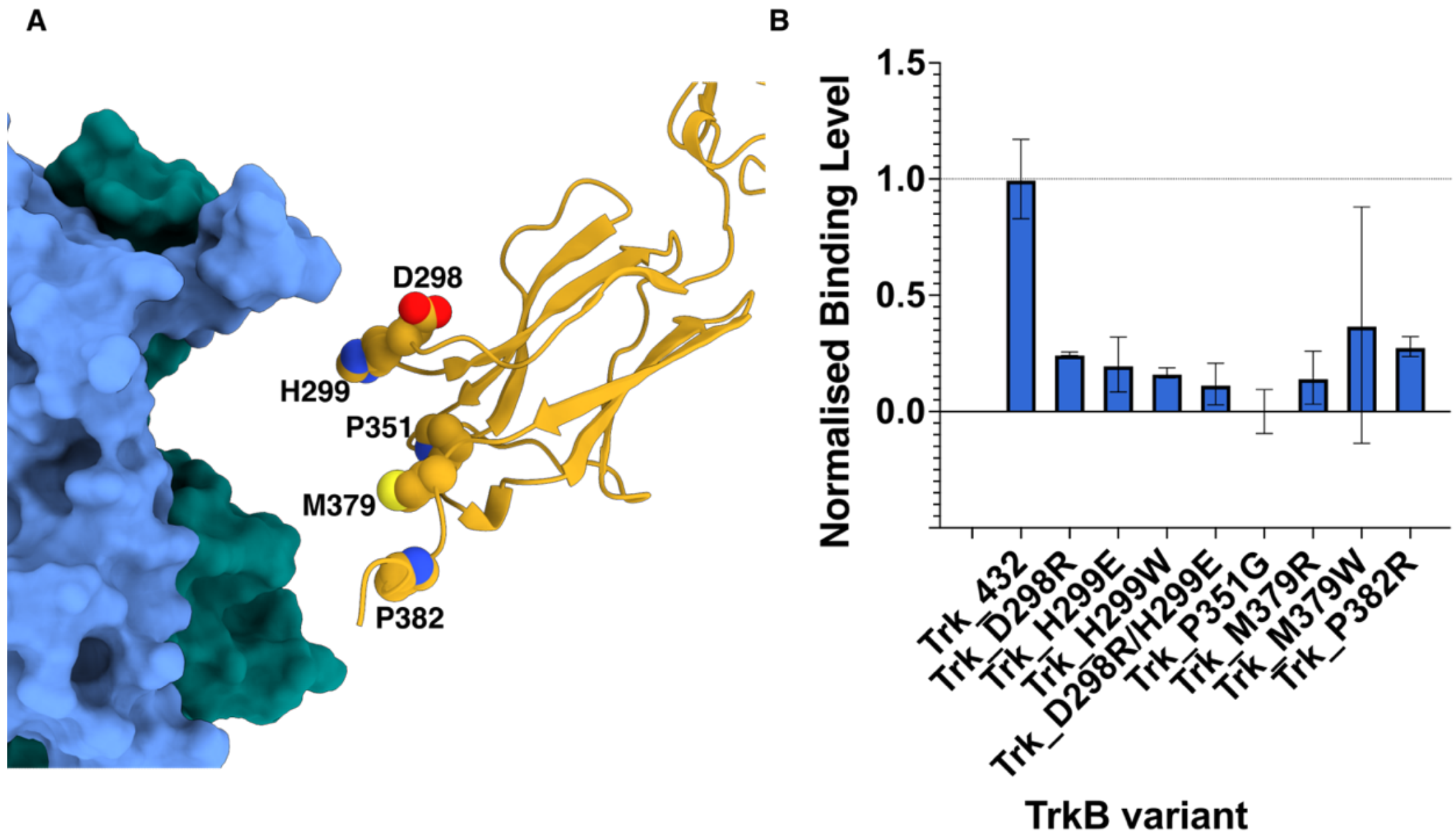

Fig. S6: Design of a BDNF-binding deficient TrkB mutant, $TrkB^{null}$. (A) The sites of chosen mutations mapped onto the $TrkB^{383}$:$BDNF^{mat}$ structure, (interface opened up for clarity) (B) SPR binding analysis of TrkB variants. Mean of 3 experiments ± S.D. Values are normalized to wild type $TrkB^{432}$.

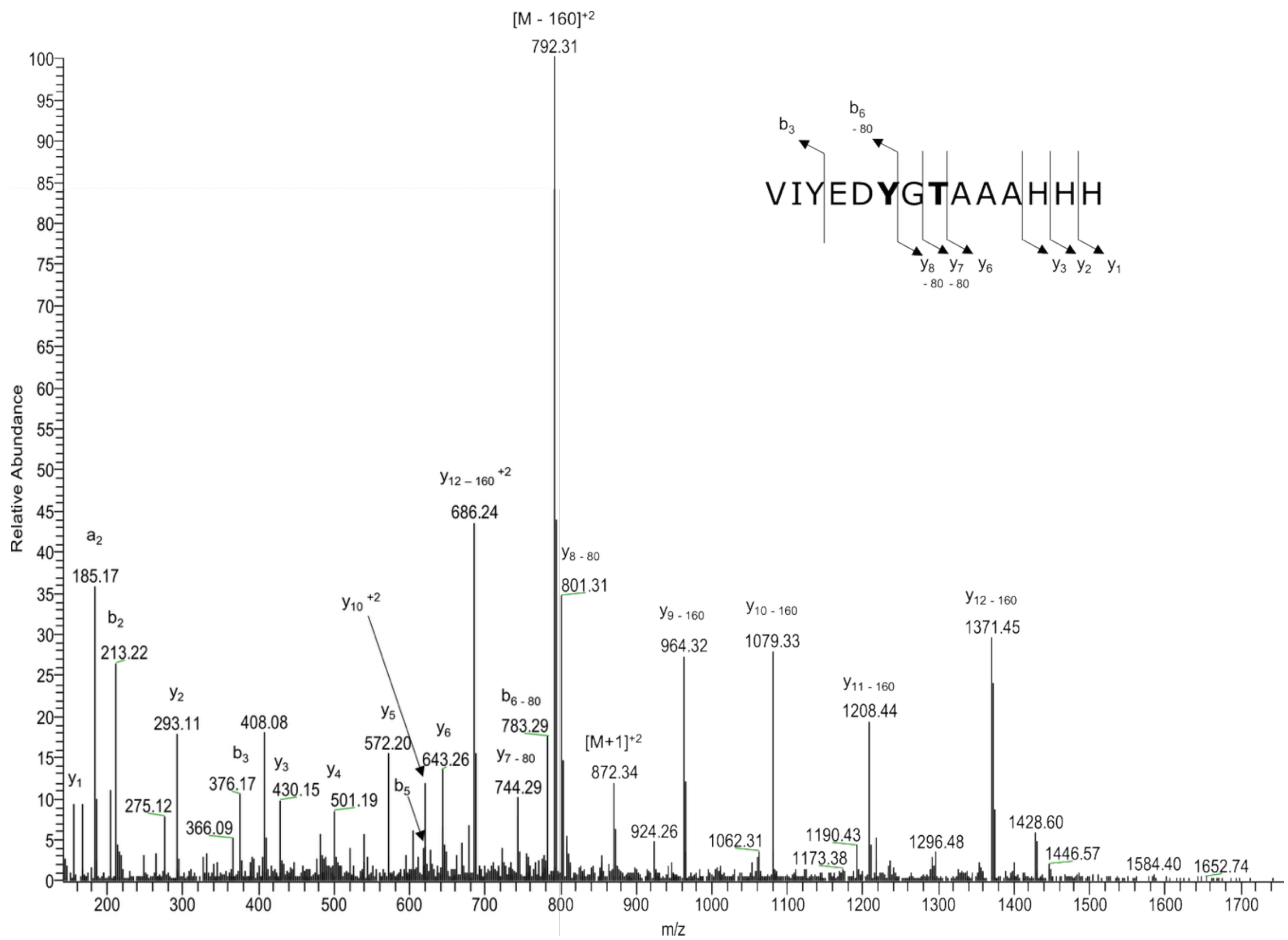

Fig. S7: Expanded view of Figure 1J. Figure shows mass spectrometry evidence for sulfotyrosine at residue tyrosine 400 of recombinant human TrkB, digested with bromelain.

Table S1.

	ZEB85 VHL Apo<br>PDB: 30PO	ZEB85 VHL Bound<br>PDB: 30PP	TrkB:BDNF complex<br>PDB: 30PU
Wavelength	0.9795 Å	0.6199 Å	0.9796 Å
Resolution range	55.28 - 1.88<br>(1.94 - 1.88)	42.59 - 2.06<br>(2.13 - 2.06)	64.19 - 3.0<br>(3.11 - 3.0)
Space group	P 64 2 2	P 64 2 2	C 1 2 1
Unit cell	a=b=110.56 Å<br>c= 184.30 Å<br>90° 90° 120°	a=b= 110.96 Å<br>c= 184.01 Å<br>90° 90° 120°	a= 56.88 Å<br>b=179.73 Å c=183.45 Å<br>90° 90.04° 90°
Total reflections	6541514 (609132)	3194219 (323105)	506973 (45341)
Unique reflections	54723 (5352)	42061 (4109)	36805 (3618)
Multiplicity	119.5 (113.8)	75.9 (78.6)	13.8 (12.5)
Completeness (%)	99.93 (100.00)	99.72 (99.47)	69.96 (25.06)<br>(*STARANISO processed*)
Mean I/sigma(I)	21.55 (0.31)	11.80 (0.59)	6.35 (0.09)
Wilson B-factor	48.46	47.87	107.63
R-pim	0.01209 (0.6188)	0.02689 (0.3572)	0.05366 (4.401)
CC1/2	1 (0.425)	1 (0.316)	0.982 (0.179)
CC*	1 (0.772)	1 (0.693)	0.995 (0.552)
Reflections used in refinement	54687 (5352)	42039 (4109)	25738 (912)
Reflections used for R-free	2669 (270)	2102 (201)	1349 (42)
R-work	0.1934 (0.3914)	0.2136 (0.3887)	0.2531 (0.4131)
R-free	0.2317 (0.4213)	0.2494 (0.3766)	0.2835 (0.4426)
Number of non-hydrogen atoms	3803	3844	7409
macromolecules	3550	3714	7311
ligands	94	71	98
solvent	207	101	0
Protein residues	459	478	936
RMS(bonds)	0.012	0.003	0.003
RMS(angles)	1.09	0.61	0.61
Ramachandran favored (%)	96.45	96.09	92.53
Ramachandran allowed (%)	3.55	3.91	7.14
Ramachandran outliers (%)	0.00	0.00	0.32
Rotamer outliers (%)	0.26	0.25	0.84
Clashscore	4.11	2.60	3.83
Average B-factor	61.09	63.62	106.01
Macromolecules	61.02	63.82	105.38
Ligands	69.46	68.22	152.85
Solvent	60.38	55.20	N/A

X-ray Crystallographic data and refinement statistics
Statistics for the highest-resolution shell are shown in parentheses.

Table S2

**Immobilised Partner**	**Immobilisation method**	**Analyte**	**K_D***
**TrkB_383**	Amine Coupling	ZEB85	N.B
**TrkB_432**	Amine Coupling	ZEB85	0.24 nM ± 0.08 nM
**TrkB_383-432 (Y400)**	Biotin/Streptavidin	ZEB85	2.34 µM ± 0.91 µM
**TrkB_393-405 (Y400)**	Biotin/Streptavidin	ZEB85	N.B
**TrkB_395-402 (pY400)**	Biotin/Streptavidin	ZEB85	N.B
**TrkB_395-402 (sY400)**	Biotin/Streptavidin	ZEB85	0.52 nM ± 0.71 nM
**BDNF**	Amine Coupling	TrkB_432	9.8 nM ± 6.24 nM
**BDNF**	Amine Coupling	TrkB_421	12.1 nM ± 1.15 nM
**BDNF**	Amine Coupling	TrkB_412	3.8 nM ± 0.49 nM
**BDNF**	Amine Coupling	TrkB_402	9.1 nM ± 0.19 nM
**BDNF**	Amine Coupling	TrkB_393	37.7 nM ± 32.5 nM
**BDNF**	Amine Coupling	TrkB_383	35.7 nM ± 18.4 nM
**BDNF**	Amine Coupling	TrkB_432_D298R_H299E	N.B
**BDNF**	Amine Coupling	TrkB_432_Y400F	15.3 nM ± 6.6 nM

Summary of Surface Plasmon resonance data

*****Geometric mean ± 0.5x range, all SPR experiment n=3 or greater.

N.B = no binding detected.

SI References

1. O. Perisic, P. A. Webb, P. Holliger, G. Winter, R. L. Williams, Crystal structure of a diabody, a bivalent antibody fragment. *Structure* **2**, 1217–1226 (1994).

2. P. Holliger, T. Prospero, G. Winter, “Diabodies”: small bivalent and bispecific antibody fragments. *Proc Natl Acad Sci U S A* **90**, 6444–6448 (1993).

3. T. R. Sosnick, D. C. Benjamin, J. Novotny, P. A. Seeger, J. Trewhella, Distances between the antigen-binding sites of three murine antibody subclasses measured using neutron and x-ray scattering. *Biochemistry* **31**, 1779–1786 (1992).

4. D. Pulido, S.-A. Hussain, E. Hohenester, Crystal Structure of the Heterotrimeric Integrin-Binding Region of Laminin-111. *Structure* **25**, 530–535 (2017).

5. D. Soppet, *et al.*, The neurotrophic factors brain-derived neurotrophic factor and neurotrophin-3 are ligands for the trkB tyrosine kinase receptor. *Cell* **65**, 895–903 (1991).

6. S. P. Squinto, *et al.*, trkB encodes a functional receptor for brain-derived neurotrophic factor and neurotrophin-3 but not nerve growth factor. *Cell* **65**, 885–893 (1991).

7. D. N. Perkins, D. J. C. Pappin, D. M. Creasy, J. S. Cottrell, Probability-based protein identification by searching sequence databases using mass spectrometry data. *Electrophoresis* **20**, 3551–3567 (1999).

8. A. Keller, A. I. Nesvizhskii, E. Kolker, R. Aebersold, Empirical Statistical Model To Estimate the Accuracy of Peptide Identifications Made by MS/MS and Database Search. *Anal. Chem.* **74**, 5383–5392 (2002).

9. G. Winter, *et al.*, DIALS: implementation and evaluation of a new integration package. *Acta Cryst D* **74**, 85–97 (2018).

10. A. J. McCoy, *et al.*, Phaser crystallographic software. *J Appl Cryst* **40**, 658–674 (2007).

11. D. Li, *et al.*, A potent human neutralizing antibody Fc-dependently reduces established HBV infections. *eLife* **6**, e26738 (2017).

12. P. Emsley, B. Lohkamp, W. G. Scott, K. Cowtan, Features and development of Coot. *Acta Crystallogr D Biol Crystallogr* **66**, 486–501 (2010).

13. F. Gorrec, The MORPHEUS protein crystallization screen. *J Appl Cryst* **42**, 1035–1042 (2009).

14. T. Wehrman, *et al.*, Structural and Mechanistic Insights into Nerve Growth Factor Interactions with the TrkA and p75 Receptors. *Neuron* **53**, 25–38 (2007).

15. A. A. Lebedev, M. N. Isupov, Space-group and origin ambiguity in macromolecular structures with pseudo-symmetry and its treatment with the program Zanuda. *Acta Cryst D* **70**, 2430–2443 (2014).

16. STARANISO anisotropy & Bayesian estimation server. Available at: https://staraniso.globalphasing.org/cgi-bin/staraniso.cgi [Accessed 2 April 2025].

17. E. C. Meng, *et al.*, UCSF ChimeraX: Tools for structure building and analysis. *Protein Science* **32**, e4792 (2023).

18. C. D. Harvey, *et al.*, A genetically encoded fluorescent sensor of ERK activity. *Proc Natl Acad Sci U S A* **105**, 19264–19269 (2008).

19. S. Ateaque, *et al.*, Selective activation and down-regulation of Trk receptors by neurotrophins in human neurons co-expressing TrkB and TrkC. *Journal of Neurochemistry* **161**, 463–477 (2022).

20. R. P. Rambo, J. A. Tainer, Information theory optimization of signals from small-angle scattering measurements. *Biophysical Journal* **124**, 2511–2522 (2025).

21. D. I. Svergun, Restoring Low Resolution Structure of Biological Macromolecules from Solution Scattering Using Simulated Annealing. *Biophysical Journal* **76**, 2879–2886 (1999).

22. K. Manalastas-Cantos, *et al.*, *ATSAS 3.0* : expanded functionality and new tools for small-angle scattering data analysis. *J Appl Crystallogr* **54**, 343–355 (2021).
